# Bioengineered embryoids mimic post-implantation development in vitro

**DOI:** 10.1038/s41467-021-25237-8

**Published:** 2021-08-26

**Authors:** Mehmet U. Girgin, Nicolas Broguiere, Sylke Hoehnel, Nathalie Brandenberg, Bastien Mercier, Alfonso Martinez Arias, Matthias P. Lutolf

**Affiliations:** 1 Laboratory of Stem Cell Bioengineering, Institute of Bioengineering, School of Life Sciences, Ecole Polytechnique Fédérale de Lausanne (EPFL), Lausanne, Switzerland; 2 SUN bioscience, EPFL Innovation Park, Lausanne, Switzerland; 3 Faculty of Medicine and Pharmacy, University of Grenoble Alpes, Grenoble, France; 4 Department of Genetics, University of Cambridge, Cambridge, UK; 5 Institute of Chemical Sciences and Engineering, School of Basic Sciences, Ecole Polytechnique Fédérale de Lausanne (EPFL), Lausanne, Switzerland; 6 Roche Institute for Translational Bioengineering (ITB), Pharma Research and Early Development (pRED), Roche Innovation Center Basel, Basel, Switzerland; 7 Present Address: Biozentrum, University of Basel, 4056 Basel, Switzerland

**Keywords:** Biological models, Embryology, Embryonic stem cells, Stem-cell differentiation, Biomedical engineering

## Abstract

The difficulty of studying post-implantation development in mammals has sparked a flurry of activity to develop in vitro models, termed embryoids, based on self-organizing pluripotent stem cells. Previous approaches to derive embryoids either lack the physiological morphology and signaling interactions, or are unconducive to model post-gastrulation development. Here, we report a bioengineering-inspired approach aimed at addressing this gap. We employ a high-throughput cell aggregation approach to simultaneously coax mouse embryonic stem cells into hundreds of uniform epiblast-like aggregates in a solid matrix-free manner. When co-cultured with mouse trophoblast stem cell aggregates, the resulting hybrid structures initiate gastrulation-like events and undergo axial morphogenesis to yield structures, termed *EpiTS embryoids*, with a pronounced anterior development, including brain-like regions. We identify the presence of an epithelium in EPI aggregates as the major determinant for the axial morphogenesis and anterior development seen in *EpiTS embryoids*. Our results demonstrate the potential of *EpiTS embryoids* to study peri-gastrulation development in vitro.

## Abstract (web summary)

Previous approaches to derive embryoids either lack physiological morphology and signaling interactions, or are unconducive to model post-gastrulation development. Here the authors use a high-throughput approach to induce mouse embryonic stem cells into epiblast-like aggregates, which are then co-cultured with mouse trophoblast stem cell aggregates, to yield embryoids with axial morphogenesis and anterior development.

## Introduction

Recapitulating early mammalian development in vitro is an important challenge that could overcome the experimental bottleneck created by intrauterine development and assist in the reduction of the use of animals in research^1^. The control of the differentiation potential of embryonic stem cells (ESCs) has been a central feature in these efforts. Constraining the differentiating cells within micropatterned substrates controls the stochastic differentiation of cells in adherent culture and results in patterns that resemble the organization of germ layers in the embryo^2,3^. Three-dimensional embryoid models, trigger self-organization programs that mimic much of the early development of the embryo and overcome the heterogeneous and heterochronic patterns characteristic of Embryoid Bodies (EBs)^4^. In particular, gastruloids^5^, aggregates of defined numbers of ESCs, develop derivatives of all germ layers with spatiotemporal patterns characteristic of embryos even though they lack clear brain structures^6^. Gastruloids have proven useful tools to explore the consequences of gastrulation in the absence of extraembryonic tissues, such as formation of cardiac primordia^7^ or somite-like structures^8,9^. Other embryoid models have explored interactions between embryonic and extraembryonic tissues during early development by taking advantage of the existence of trophoblast (TSC) and extraembryonic endoderm (XEN) stem cells to recreate the early conceptus. Co-culture of TSCs and XEN cells with mouse ESCs in Matrigel results in a suite of structures, including blastoids^10^, ETS^11^- and ETX^12^-embryos that recapitulate events and interactions of the pre-gastrulation embryo. A challenge with ETS- and ETX-embryos is the reliance on the self-organizing activity of the cellular compartments that results in their stochastic occurrence; only ~20% of the starting aggregates will form patterned structures^11^, which limits their broader applicability. Furthermore, their developmental potential is not yet clear, as there are no reports of their development beyond early gastrulation stages^11^. Altogether, these pioneering in vitro models of early embryo development highlight the remarkable capacity of embryonic and extraembryonic cells to organize themselves but thus far have not yet been ideal to explore the fate and derivatives of cells in the emerging structures.

Here, we used a bioengineering approach combining features of ETS embryos and gastruloids to build a standardized embryoid model that could capture the events leading to and resulting from gastrulation. We hypothesized that to achieve this, it would be essential to optimize the starting culture conditions in multicellular aggregates to assemble, in the same embryoid, an anterior neuroepithelium with a posterior *T/Bra*-expressing cell ensemble. We used arrays of cell-repellent hydrogel microwells to separately generate epithelialized or non-epithelialized epiblast-like (EPI) aggregates, as well as trophoblast stem cell (TSC) aggregates mimicking extraembryonic ectoderm, in a highly scalable manner. When assembled together in low-attachment wells in serum-free medium, EPI and TSC aggregates rapidly merged and underwent symmetry breaking similar to gastrulating embryos, as demonstrated by polarized and restricted *Brachyury (T/Bra)* expression. Importantly, in these structures, *T/Bra* expression dynamics was strictly dependent on the epithelial architecture of the EPI aggregates. Subsequently, hybrid EPI/TSC structures, termed *EpiTS* embryoids, underwent axial morphogenesis to display patterning along anterior–posterior, dorsal–ventral, and medio-lateral axes. Strikingly, we observed that embryoids formed from non-epithelialized EPI aggregates predominantly generated mesendodermal tissue, whereas epithelialized ones formed cell types that are present in the developing midbrain/hindbrain. Our approach enables the generation of various tissues in a stereotyped and highly scalable manner, with independent modulation of physical (e.g., size, epithelial architecture) and biological (e.g., provision of signaling molecules) properties of EPI and TSC aggregates, such as to systematically parse out the role of these parameters in promoting key steps in embryogenesis.

## Results

### Scalable formation of EPI aggregates

**Fig. 1:**
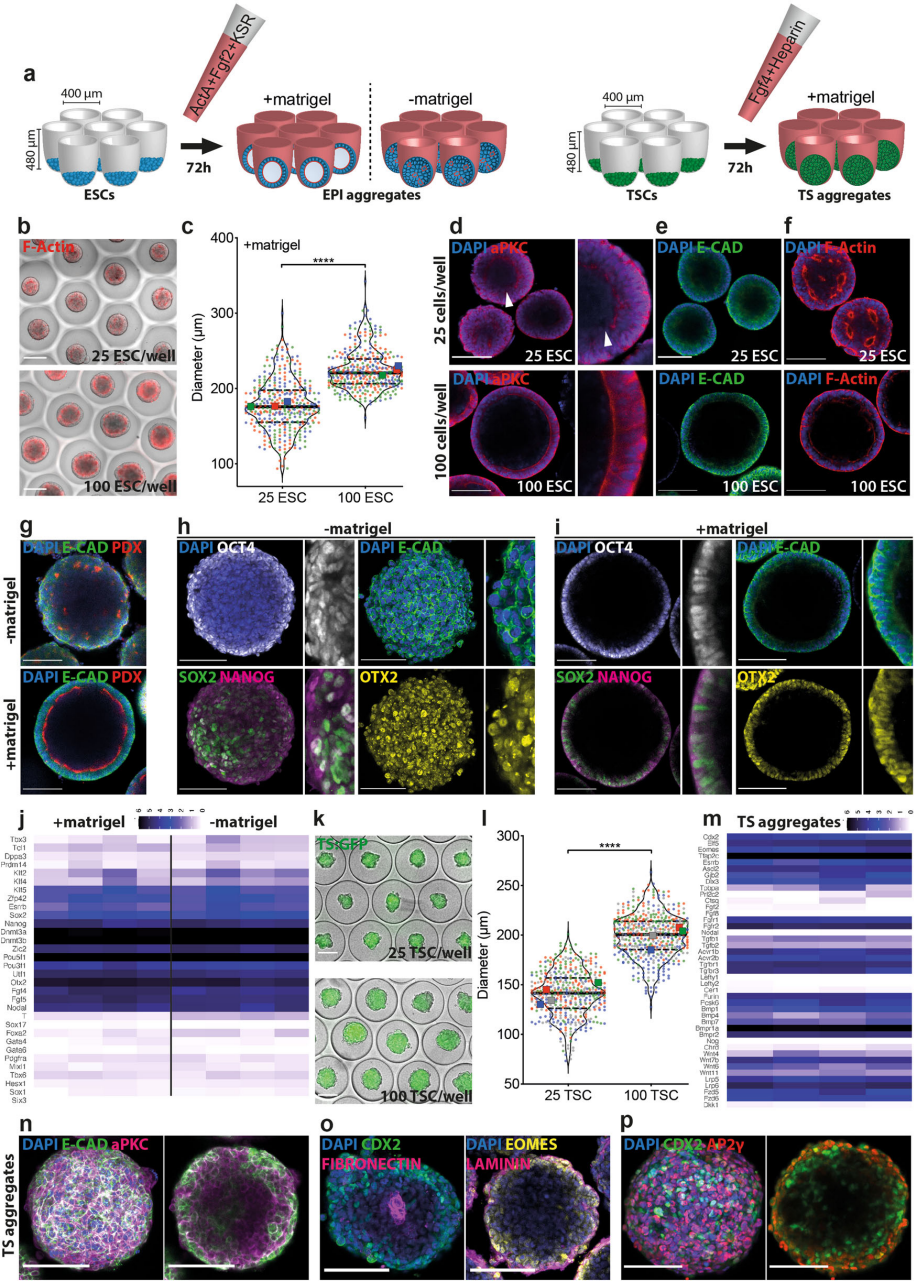
Formation and characterization of EPI and TS aggregates. **a** Schematic representation of the workflow showing aggregation of ESCs and TSCs on PEG microwells. **b** Representative images showing EPI aggregates on microwells at 72 h formed from 25 cells/well (top) or 100 cells/well (bottom) showing F-actin staining by phalloidin. Scale bars: 200 µm. **c** Comparing minimum ferret diameters of EPI aggregates at 72 h formed from 25 or 100 cells/well. For 25 ESC and 100ESC conditions, total number of embryoids analyzed were 362 and 361, respectively. Data are collected from three biologically independent experiments. Large symbols indicate mean values of each replicate. Black lines indicate median and quartiles. **d**–**f** Confocal images of EPI aggregates formed from 25 cells/well or 100 cells/well fixed at 72 h and stained for *aPKC* (**d**)*, E-cadherin* (**e**), and *F-actin* by phalloidin (**f**). Nuclei were stained with DAPI. Note multilayered epithelium in EPI aggregates formed from 25 cells/well showing discontinuous *aPKC* staining (white arrows) compared to single-layer epithelium in EPI aggregates formed from 100 cells/well showing continuous *aPKC* signal. Scale bars: 100 µm. **g** Confocal images of aggregates formed from 100 cells/well with or without Matrigel fixed at 72 h and stained for *E-cadherin* and *Podocalxyin*. Nuclei were stained with DAPI. Scale bars: 100 µm. **h**, **i** Confocal images of EPI aggregates formed from 100 cells/well without (**h**) or with (**i**) Matrigel, fixed at 72 h showing expression of *Otx2*, *Oct4*, *Sox2*, *Nanog*, and *E-cadherin*. Nuclei were stained with DAPI. Scale bars: 100 µm. **j** Bulk RNA-sequencing analysis of epithelialized and non-epithelialized EPI aggregates at 72 h formed from 100 cells/well, showing expression levels of pluripotency, epiblast-specific and early differentiation genes. Data are collected from four biologically independent experiments. **k** Representative images showing TS aggregates on microwells at 72 h formed from 25 cells/well (top) or 100 cells/well (bottom) showing ubiquitous GFP expression. Scale bars: 200 µm. **l** Comparing minimum ferret diameters of TS aggregates at 72 h formed from 25 or 100 cells/well. For 25 TSC and 100TSC conditions, total number of embryoids analyzed were 455 and 478, respectively. Data are collected from four biologically independent experiments. Large symbols indicate mean values of each replicate. Black lines indicate median and quartiles. **m** Bulk RNA-sequencing analysis of TS aggregates at 72 h formed from 100 cells/well, showing expression levels of stem cell and differentiation markers as well as key pathway agonists/antagonists. Data are collected from four biologically independent experiments. **n**–**p** Confocal images of TS aggregates formed from 100 cells/well, fixed at 72 h showing expression of *E-cadherin* and *aPKC* (**n**), *Cdx2, Fibronectin, Laminin*, and *Eomes* (**o**), *Cdx2* and *Ap2γ* (**p**). Nuclei were stained with DAPI. Scale bars: 100 µm. For statistical analysis, two-tailed unpaired Student’s *t*-test (**c**, **l**) or multiple *t*-tests followed by Holm-Sidak multiple comparison test (**j**, **m**) were performed. For **j**, False Discovery Rate (FDR) with cutoff *p* value <0.5 was used to test significant differences between +matrigel and -matrigel conditions. Following *P* value style was used: (****) < 0.0001, (***) 0.0002, (**) 0.0021, (*) 0.0332, (ns) 0.1234. Source data are provided as a Source Data file.

We aggregated ESCs in round-bottom microwell arrays composed of nonadhesive PEG hydrogels^13^ in epiblast induction medium comprising Activin-A, bFGF, and KSR, supplemented with low percentage (3%) Matrigel to induce epithelialization^14^ (Fig. 1a). The microwells allowed us to titrate the average number of cells seeded in each well, resulting in aggregates of defined size (Fig. 1b, Supplementary Fig. 1a, upper panel). The starting number of cells determined the size of the aggregate at 72 h of culture: an average of ~25 ESCs per microwell yielded aggregates with ~180 µm diameter, whereas seeding ~100 cells reached a diameter of ~230 µm (Fig. 1c). These aggregates featured a single lumen surrounded by an *E-cadherin*-positive polarized epithelium, displaying apical *Pdx* and *Par6* expression (Fig. 1d–f, lower panel; Supplementary Fig. 1c). Smaller aggregates exhibited a discontinuous apical expression of *aPKC* surrounded by a multilayered *E-cadherin*-positive epithelium and multiple *F-actin-*labeled cavities, suggesting poor epithelialization and incomplete lumenization (Fig. 1d–f, upper panel). These results demonstrate that a critical aggregate size needs to be reached to form an apicobasally polarized epithelium with a central lumen, a phenomenon reminiscent of epiblast maturation^15^. When cells were aggregated in the absence of Matrigel, we detected increased cell shedding (Supplementary Fig. 1a, lower panel, white arrows) but no significant difference in aggregate diameter (Supplementary Fig. 1b). However, these aggregates composed of *E-cadherin*-positive cells demonstrated inverted *F-actin* polarity and featured multiple *Pdx*-positive and *Par6*-positive foci with no clear epithelium or lumen (Fig. 1g, Supplementary Fig. 1d). These results show that polarized epithelial aggregates of defined size can be readily generated from ESCs using hydrogel microwell arrays, and confirmed that the provision of Matrigel is critical for their lumenization and epithelialization^16^.

Of note, affecting cytoskeletal activity by inhibiting stress fiber formation (Y-27632) and disrupting the actin network (CK-666) resulted in loss of a continuous apicobasally polarized epithelium. Interestingly, inducing stress fiber formation with LPA did not have any effect on epithelialization (Supplementary Fig. 2a, b). Moreover, blocking actin-myosin interaction (Blebbistatin), myosin activity (ML-7 and Calcyculin-A), or actin polarization dynamics (Cytochalasin-D/Latrunculin-A and Jasplakinolide) prevented formation of compact aggregates and, in some cases (ML-7 and Cyto-D), epithelialization. Interestingly, treatment with the *PTEN* inhibitor BpV showed a similar effect, in line with previous reports highlighting the role of *PTEN* in epiblast polarization^17^. These results suggested that actin polymerization and actin-myosin interaction play crucial roles in the polarization and epithelialization of EPI aggregates.

To test the identity of the aggregates after 72 h, we performed immunostaining for pluripotency and epiblast markers. In the absence of Matrigel, the aggregates showed a uniform expression of *Otx2* and *E-cadherin* (Fig. 1h). Interestingly, *Sox2* expression was detected in a polarized fashion in cells that showed high WNT and low TGF-β activity (Fig. 1h, Supplementary Fig. 3a), likely marking the remaining naïve pluripotent stem cells^18^, suggesting establishment of an naïve-to-primed axis. Epithelialized aggregates were comprised of *E-cadherin+* cells organized in a columnar epithelium and were uniformly positive for *Oct4* and *Otx2*. Expression of *Sox2* and *Nanog* were detected at lower levels, often in a salt-and-pepper fashion. In general, *Sox2+* cells in epithelialized aggregates demonstrated high WNT and low TGF-β activity (Fig. 1i, Supplementary Fig. 3b). Bulk RNA sequencing revealed low expression levels for naïve pluripotency factors *Klf2/4*, *Dppa3*, *Tbx3* and higher levels of epiblast-specific genes *Fgf5*, *Otx2, Utf1* in EPI aggregates. At this stage, transcripts that mark further differentiated states such as *T, Sox1*, or *Sox17* were not upregulated albeit the levels were higher in non-epithelialized EPI aggregates (Fig. 1j, Supplementary Fig. 3c). Altogether, these observations suggested that ESCs could be coaxed into EPI aggregates that morphologically (i.e., lumenized/epithelialized) and transcriptionally resemble post-implantation epiblast.

### Scalable formation of TSC aggregates

In the early embryo, the epiblast develops in conjunction with extraembryonic tissues, and signaling from the extraembryonic ectoderm is involved in specifying the onset of gastrulation^19^. To mimic these interactions, we used the same hydrogel microwell array technology to generate TSC aggregates (Fig. 1k). On average, TSC aggregates were slightly smaller than EPI aggregates but their size at 72 h was also found to be dependent on the initial cell seeding concentration: aggregates composed of 25 cells or 100 cells reached a diameter of ~140 or ~200 µm, respectively (Fig. 1l). We detected expression of *Cdx2*, *Elf5*, and *Eomes* transcripts and high levels of *Tfap2c* (Fig. 1m), suggesting that aggregated TSCs maintain stem cell identity and get primed for differentiation^20^. At this timepoint, genes expressed in more differentiated cell types such as spongiotrophoblast or giant cells were detected at relatively low levels (Fig. 1m), demonstrating that TSC aggregates have acquired an intermediate state, likely corresponding to extraembryonic ectoderm^21^. Importantly, TSC aggregates expressed several receptors for FGF and TGF-β pathways but not many of the ligands, in line with previous reports suggesting epiblast as source of Fgf4 and Nodal for embryonic development^22^. For BMP and WNT pathways, we could detect both receptor and ligand expression in TSC aggregates^23^.

Unlike EPI aggregates, TSC aggregates grown in the presence of low percentage Matrigel did not lumenize (Fig. 1n). Immunostaining showed that TSC aggregates could interact with the surrounding *laminin*-based extracellular matrix (ECM) and deposited *fibronectin* at the aggregate core (Fig. 1o). Expression of the stem cell markers *Cdx2*, *Tfap2c*, and *Eomes* were primarily maintained on the periphery, suggesting initiation of differentiation from the core of the TSC aggregates^24^ (Fig. 1o, p).

### Formation of EpiTS embryoids

**Fig. 2:**
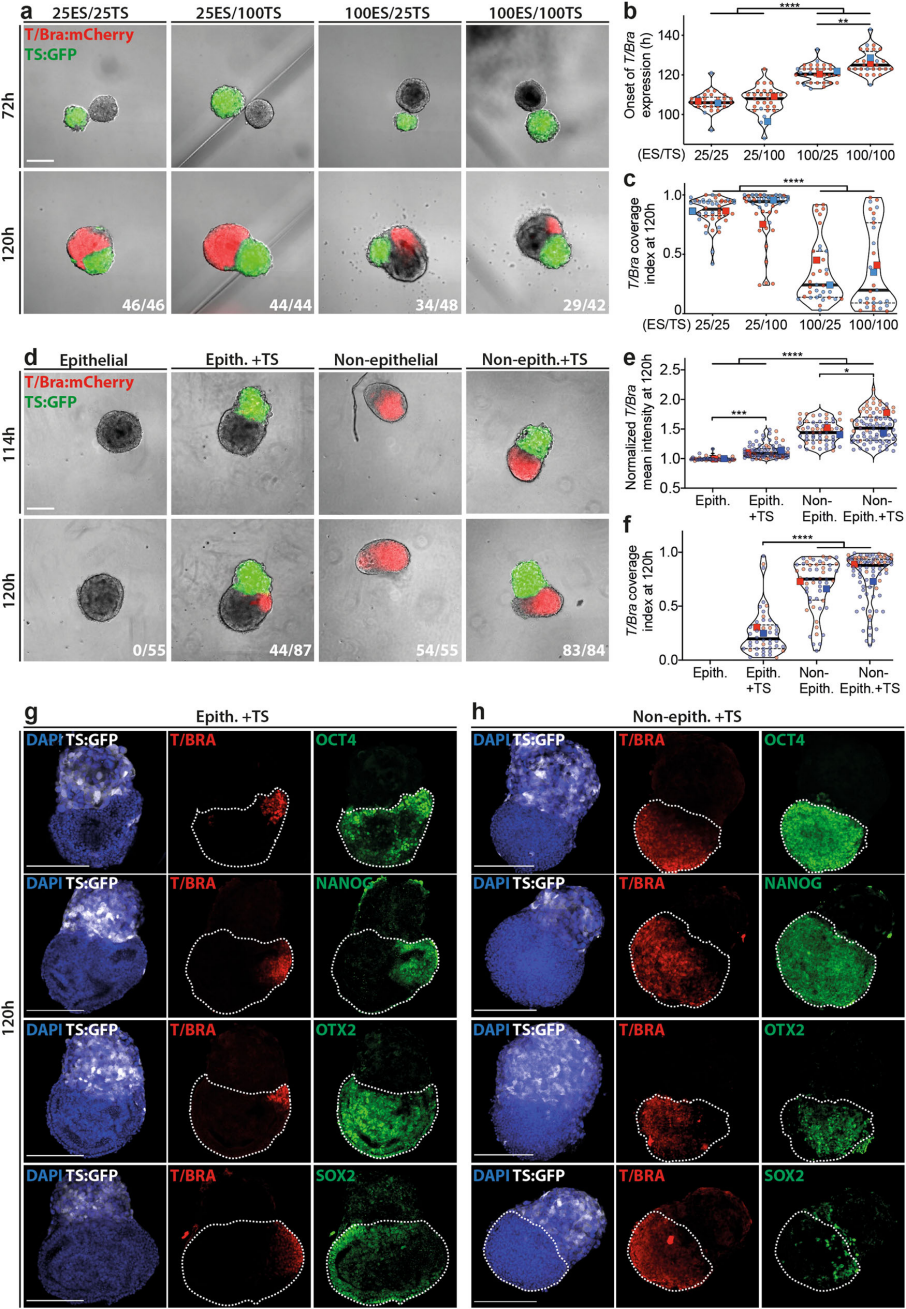
Effects of size and epithelial architecture of EPI aggregates on *T/Bra* expression dynamics in *EpiTS* embryoids. **a** Representative images showing *T/Bra* expression dynamics in *EpiTS* embryoids formed from different starting cell numbers per well (ESC/TSC). **b** Timelapse analysis between 78 h and 149 h with 2 h interval showing the onset of *T/Bra* expression. For 25/25, 25/100, 100/25, and 100/100 conditions, total number of embryoids analyzed were 26, 27, 31, and 26, respectively. Data are collected from two biologically independent experiments. Adjusted *p* values are: 100/25 vs. 100/100 *p* = 0.0013*.* **c** Coverage index of *T/Bra* expression calculated by division of *T/Bra*-positive area to EPI area. Data were collected from two biologically independent experiments. **d**–**f** Representative images at 114 h and 120 h showing *T/Bra* expression (**d**), background normalized mean intensity (**e**), and coverage (**f**) in epithelialized (+matrigel) or non-epithelialized (-matrigel) EPI aggregates, cultured in the presence or absence of TS aggregates at 120 h. For **e**, adjusted p values are: non-epith. vs non-epith.+TS *p* = 0.0384; Epith. vs Epith.+TS *p* = 0.0001. Data were collected from two biologically independent experiments. **g**, **h** Representative confocal images at 120 h showing *T/Bra, Oct4, Nanog, Otx2,* and *Sox2* expression in epithelialized (**g**) or non-epithelialized (**h**) embryoids formed from 100ESC/100TSC condition. Nuclei were stained with DAPI. GFP-labeled TS cells were depicted in white. Dashed lines indicate EPI compartment. For all conditions in **a**–**c** and **d**–**f**, number of *T/Bra*:mCherry-positive embryoids over total number of embryoids analyzed are indicated at bottom right of **a** and **d**, respectively. Large symbols indicate mean values of each replicate. Black lines indicate median and quartiles. For all statistical analysis, one-way ANOVA followed by Tukey multiple comparison test was performed. Following *P* value style was used: (****) < 0.0001, (***) 0.0002, (**) 0.0021, (*) 0.0332, (ns) 0.1234. Scale bars: 200 µm. Source data are provided as a Source Data file.

Next, we assembled the EPI and TSC aggregates into structures that we termed *EpiTS* embryoids. Individual EPI and TSC aggregates were transferred at 72–75 h to U-bottom low-attachment wells of a 96-well plate, where they fused together within a few hours (Fig. 2a, top panel). Time-lapse imaging revealed that by 120 h, *T/Bra* expression appeared at the aggregate interface. The size of EPI and TSC aggregates strongly influenced the timing of *T/Bra* expression (Fig. 2b). Embryoids composed of smaller EPI aggregates initiated *T/Bra* expression generally before 110 h; by 120 h, all embryoids had *T/Bra*-expressing cells regardless of the size of TSC aggregates they were fused to (Fig. 2a, bottom panel). On the other hand, *EpiTS* embryoids formed from larger EPI aggregates showed a delayed onset of *T/Bra* expression (Fig. 2b), with 50–70% being *T/Bra*-positive at 120 h (Fig. 2a, bottom panel). In addition to differences in timing, we observed a strong dependence of the initial EPI aggregate size on the *T/Bra* expression domain (Fig. 2c). At 120 h, embryoids formed from smaller EPI aggregates acquired a dispersed expression of *T/Bra*, covering almost the entire EPI compartment, whereas *EpiTS* embryoids formed from bigger EPI aggregates featured a restricted *T/Bra* expression (Supplementary Movies 1–4).

To test whether the appearance of *T/Bra* expression was dependent on the epithelial architecture of the aggregates, or the presence of an extraembryonic compartment, we generated epithelialized (with Matrigel) and non-epithelialized (w/o Matrigel) EPI aggregates, and cultured them in the presence or absence of TSC aggregates. Almost all non-epithelialized EPI aggregates displayed *T/Bra* expression by 120 h, independent of the extraembryonic compartment. In contrast, epithelialized EPI aggregates alone did not initiate *T/Bra* expression and required co-culture with TSC aggregates (Fig. 2d, e), similar to embryo explants cultured without extraembryonic ectoderm^25^. Moreover, the *T/Bra* expression domain was found to be more restricted in epithelialized embryoids (Epith.+TS) compared to non-epithelialized (Non-epith.+TS) ones (Fig. 2f).

To test whether *T/Bra* induction is specific to TSC aggregates and not due to any cell–cell contact, we co-cultured epithelialized EPI aggregates with aggregates composed of mouse embryonic fibroblasts (MEFs). MEF aggregates could occasionally induce *T/Bra* expression, albeit at much lower levels compared TSC aggregates (Supplementary Fig. 4a, b). This suggests that cellular interaction alone is not sufficient to induce *T/Bra* expression and that TSC aggregate-specific signaling may be necessary.

In vivo, signaling from the extraembryonic ectoderm is crucial for the initiation of gastrulation^26^. To better understand the signaling influence of the TSC compartment in our embryoids, we replaced the cellular aggregates with cell-adhesive hydrogel microbeads (composed of denatured collagen cross-linked to dextran) coated with various (diffusible) morphogens involved in early mouse development. When coupled to EPI aggregates at 72 h, plain beads or beads coated with Fgf2, Activin-A, or Wnt3a could induce *T/Bra* expression, although at lower levels than TSC aggregates. Strikingly, Bmp4-coated beads reached the highest *T/Bra* expression level, outperforming the TSC aggregates (Supplementary Fig. 4a, b). Similarly, Bmp4 delivery increased WNT activity, even to higher levels compared to Wnt3a (Supplementary Fig. 4a, c). Notably, we did not detect any significant change in TGF-β activity for the proteins tested (Supplementary Fig. 4a, d). Transwell co-culture of epithelialized EPI aggregates with varying numbers of TSC aggregates did not induce *T/Bra* expression (Supplementary Fig. 4c, d), showing that physical interactions between the two cell compartment is crucial for symmetry breaking. Altogether, these results highlight a role of the epithelial architecture of EPI aggregates for *T/Bra* expression dynamics. EPI aggregates that are small (i.e., poorly epithelialized), or formed in the absence of Matrigel (i.e., not epithelialized), showed an extended *T/Bra* expression domain when coupled to TSC aggregates, suggesting uniform mesodermal differentiation, while in EPI aggregates the epithelium limits *T/Bra* expression to one side of the embryoids. Furthermore, TSC aggregate-specific signaling, likely via *Bmp4*, and physical EPI-TSC interaction are both important for the reproducible symmetry breaking and induction of *T/Bra* expression in epithelialized *EpiTS* embryoids.

### Cell fate assignments in *EpiTS* embryoids

Next, we performed immunostaining and confocal microscopy to characterize the tissues present in *EpiTS* embryoids at the onset of gastrulation. In the optimal culture condition that yielded the most restricted induction of *T/Bra* expression (100ESC/100TSC), we observed that the lumen collapsed by 120 h and the *E-cadherin*/*Pdx-*positive epithelium disappeared in *T/Bra*-positive posterior domain, while it remained in the anterior domain where the cells remained in epithelial rosettes with apical E-cadherin enrichment (Supplementary Fig. 5a, dashed box (i)). Moreover, we detected a local downregulation of *laminin* where some *T-Bra*/*Snai1-*double-positive cells emerged (Supplementary Fig. 5a, dashed boxes), indicating basement membrane degradation and epithelial-to-mesenchymal transition (EMT) that precede migration from the primitive streak in the embryo^27^. Immunostaining for the pluripotency marker *Oct4* revealed uniform expression in all cells of the EPI compartment, partially overlapping with *T/Bra*-positive cells. The expression domain of *Nanog* was more restricted, encircling and colocalizing with *T/Bra* expression^3^. The epiblast marker *Otx2* was detected in a majority of the cells in the embryonic domain, excluding the *T/Bra*-positive cells. Notably, *Sox2* was solely expressed opposite and excluded from the *T/Bra* domain (Fig. 2g). We believe that the *Oct4+ T-Bra+ Nanog*+ domain in these early *EpiTS* embryoids resembles the posterior end of the early to mid-streak embryo^28^. Conversely, the *Oct4+ Otx2+ Sox2+* domain marked the opposite end of the embryoids, suggesting maintenance of the anterior pluripotent epiblast. We did not detect *Sox17*-positive cells in embryoids that initiated *T/Bra* expression, but we could detect some *Dppa3*-positive cells (Supplementary Fig. 5b), likely marking primordial germ cell (PGC) progenitors^29^.

Embryoids formed from non-epithelialized EPI aggregates showed dispersed *T/Bra* expression and did not demonstrate *E-cadherin+* rosettes. Interestingly, *Pdx* expression was downregulated in the embryonic compartment. Moreover, *laminin* was completely absent and majority of cells demonstrated *Snai1* expression, suggesting an expanded EMT-active domain (Supplementary Fig. 5c). *T/Bra* expression mostly colocalized with *Oct4*/*Nanog*; however, *Otx2/Sox2* expression spanned a smaller domain compared to epithelialized embryoids, suggesting the loss of anterior neural progenitors at the expense of expanded posterior tissue (Fig. 2h). Moreover, we detected a higher number of *Sox17*-positive endoderm-like cells and *Dppa3*-positive PGC progenitors (Supplementary Fig. 5d), indicating an increased differentiation of posterior cell types.

Collectively, these results show that the epithelial architecture of the EPI aggregates directly influences the type of tissue that is created in embryoids. The presence of an epithelium favors limited *T/Bra* induction and a larger domain of anterior epiblasts that resemble E6.5 embryos. Conversely, in the absence of an epithelium in the initial EPI aggregate, the embryoids consist largely of posterior tissues and show premature differentiation towards the endoderm lineage.

### Gastrulation-like events are orchestrated by key early developmental signaling pathways

The onset of gastrulation is the output of asymmetric signaling across the post-implantation epiblast and is tightly regulated by WNT, TGF-β, and BMP signaling pathways^30^. Embryos that lack functional β-catenin^31^ or cleavable Nodal^32^ fail to upregulate *T/Bra* and are unable to undergo gastrulation. Furthermore, embryos lacking both copies of *Bmp4* are arrested in development and do not express *T/Bra*^26^. The dependence of the symmetry breaking dynamics on the epithelial architecture of *EpiTS* embryoids led us to probe the underlying signaling pathways that regulate *T/Bra* expression by generating *EpiTS* embryoids from WNT (TLC:mCherry^33,34^) and TGF-β (AR8:mCherry^35^) reporter ESC lines and by using specific pathway inhibitors.

**Fig. 3:**
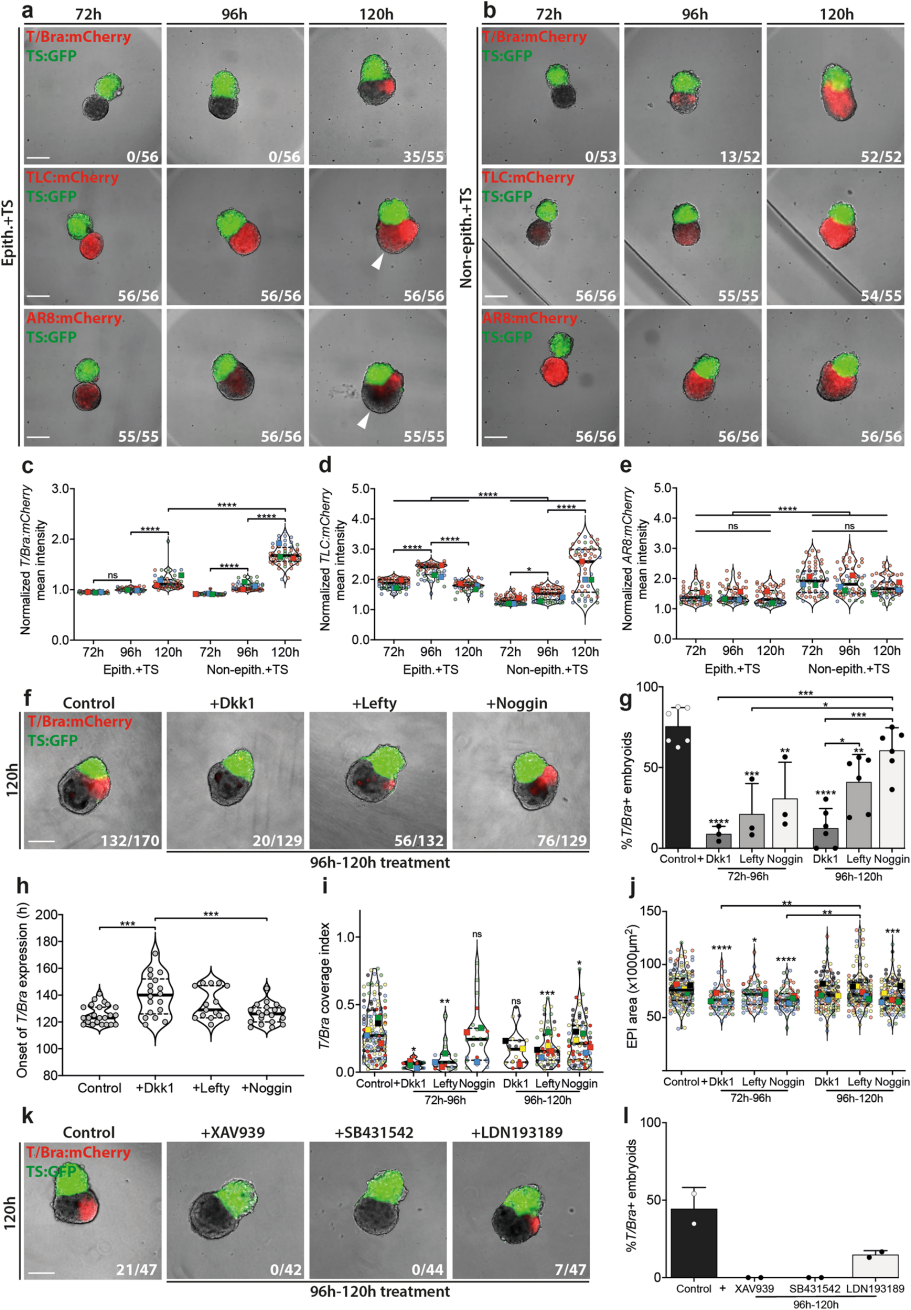
Roles of WNT, TGF-β, and BMP pathways in *T/Bra* expression dynamics. **a**, **b** Representative images showing *T/Bra:*mCherry (top), *TLC:*mCherry (middle), and *AR8:*mCherry (bottom) expression dynamics in epithelialized (**a**) and non-epithelialized (**b**) *EpiTS* embryoids between 72 and 120 h. **c**–**e** Background normalized mean intensity of *T/Bra:*mCherry (**c**), *TLC:*mCherry (**d**), and *AR8:*mCherry (**e**) in epithelialized or non-epithelialized embryoids between 72 and 120 h. Data were collected from three biologically independent experiments. For **d**, adjusted *p*-values are: Non-epith.+TS (72 h) vs Non-epith.+TS (96 h), *p* = 0.0168. **f** Representative images at 120 h showing *T/Bra:*mCherry expression in epithelialized embryoids treated with indicated inhibitors between 96 and 120 h. **g** Percentage of *T/Bra:*mCherry-positive epithelialized embryoids at 120 h, treated with indicated inhibitors between 72 and 96 h or 96 and 120 h. Background offset was used to set a threshold for *T/Bra:*mCherry expression. Adjusted *p*-values are: Control vs Lefty (96–120 h), *p* = 0.0061*;* Control vs Lefty (72–96 h), *p* = 0.0003*;* Control vs Noggin (72–96 h), *p* = 0.0033*;* Noggin (96–120 h) vs Dkk1 (96–120 h), *p* = 0.0001*;* Noggin (96–120 h) vs Lefty (72–96 h), *p* = 0.0121*;* Noggin (96–120 h) vs Dkk1 (96–120 h), *p* = 0.0001*;* Noggin (96–120 h) vs Dkk1 (72–96 h), *p* = 0.0006*;* Dkk1 (96–120 h) vs Lefty (96–120 h), *p* = 0.0325. **h** Timelapse analysis between 78 and 192 h with 2 h interval showing the onset of *T/Bra* expression. For control, Dkk1, Lefty, Noggin conditions, total number of embryoids analyzed were 23, 18, 15, 20, respectively. Data are collected from a single experiment. Adjusted *p*-values are: Control vs Dkk1, *p* = 0.0002*;* Noggin vs Dkk1, *p* = 0.0007. **i**, **j** Quantification of coverage index of *T/Bra:*mCherry expression (**i**) and EPI compartment area (**j**) at 120 h in epithelialized embryoids treated with indicated inhibitors. For **i**, adjusted *p*-values are: Control vs Dkk1 (72–96 h), *p* = 0.0123*;* Control vs Lefty (72–96 h), *p* = 0.0010*;* Control vs Lefty (96–120 h), *p* = 0.0009*;* Control vs Noggin (96–120 h), *p* = 0.0300. For **j**, adjusted *p*-values are: Control vs Lefty (72–96 h), *p* = 0.0276*;* Dkk1 (72–96 h) vs Lefty (96–120 h), *p* = 0.0049*;* Noggin (72–96 h) vs Lefty (96–120 h), *p* = 0.0021. **k** Representative images showing *T/Bra:*mCherry expression in epithelialized embryoids treated with indicated small molecule inhibitors between 96 and 120 h. **l** Percentage of *T/Bra:*mCherry-positive epithelialized embryoids at 120 h, treated with indicated inhibitors between 96 and 120 h. All embryoids were formed from 100ESC/100TSC condition. For all conditions in **a**–**e**, **f**, **g**, **i**, **j**, and **k**, **l** number of mCherry-positive embryoids over total number of embryoids analyzed are indicated at bottom right of **a**–**b**, **f**, and **k**, respectively. Large symbols indicate mean values of each replicate. Black lines indicate median and quartiles. For all statistical analysis, one-way ANOVA followed by Tukey multiple comparison test was performed. For **g**, **i**, and **j** significance was calculated in comparison to control. Following *P* value style was used: (****) < 0.0001, (***) 0.0002, (**) 0.0021, (*) 0.0332, (ns) 0.1234. Scale bars: 200 µm. Source data are provided as a Source Data file.

Time-lapse analysis showed that in non-epithelized embryoids, *T/Bra* expression begins as early as 96 h, in contrast to epithelized ones, which show a delayed initiation at 120 h (Fig. 3a–b, upper panels; Fig. 3c). In epithelialized embryoids, we could detect an increase of WNT activity from 72 to 96 h, followed by a reduction and restriction to the embryonic-extraembryonic interface by 120 h. In contrast, non-epithelial embryoids demonstrated a progressive increase in WNT activity that was detected throughout the EPI domain (Fig. 3a–b, middle panels; Fig. 3d). On the other hand, TGF-β signaling was continuously active in both conditions, but its expression domain became restricted over time. Of note, compared to epithelialized embryoids, non-epithelialized ones showed significantly higher TGF-β pathway activity from 72 to 120 h, (Fig. 3a–b, bottom panels; Fig. 3e). These results showed that in *EpiTS* embryoids, both WNT and TGF-β pathways are active and their dynamics depend on the epithelial architecture of the EPI aggregate. In epithelialized embryoids, WNT and TGF-β signaling preceded *T/Bra* induction and are colocalized by 120 h, resembling the initial primitive streak-like domain. The signaling polarization rendered the anterior of the embryoids low in WNT and TGF-β activity (Fig. 3a, white arrows, Fig. 3b), potentially preserving the potential for anterior neural progenitors^36,37^. In non-epithelialized embryoids, dispersed *T/Bra* expression was accompanied by elevated WNT and TGF-β activity by 120 h, resembling an expanded posterior domain observed in Dkk1^−/−^ and Cer1^−/−^;Lefty1^−/−^ mutant embryos^38,39^.

To test whether gastrulation-like processes observed in epithelialized *EpiTS* embryoids involve mechanisms similar to those observed in the embryo, we inhibited the above pathways by adding Dkk1 (i.e., a WNT inhibitor), Lefty (TGF-β inhibitor), and Noggin (BMP inhibitor) between 72 and 96 h or 96 and 120 h, respectively. Inhibition of WNT and TGF-β pathways in both time windows greatly reduced the number of embryoids that upregulate *T/Bra*. However, BMP inhibition by Noggin only between 72 and 96 h significantly lowered the percentage of *T/Bra*+ embryoids by 120 h (Fig. 3f, g**)**. Dkk1-treated *EpiTS* embryoids exhibited a delayed *T/Bra* expression compared to nontreated or Noggin-treated ones (median ~140 versus ~125 h) (Fig. 3h). Furthermore, we observed more restricted *T/Bra* expression in the presence of the inhibitors, which was more pronounced in the cases of Dkk1 and Lefty (Fig. 3i). Interestingly, early treatment with these inhibitors significantly reduced the total embryoid size, an effect that was more prominent when BMP signaling was inhibited (Fig. 3j). Treatment with the small molecule inhibitors XAV939 (i.e., a WNT inhibitor), SB431542 (TGF-β inhibitor), and LDN193189 (BMP inhibitor) resulted in a more pronounced reduction of *T/Bra* expression; SB431542 and XAV939 treatment led to a complete loss of *T/Bra* expression, while a small percentage of LDN193189-treated *EpiTS* embryoids still expressed *T/Bra*, yet with a domain that was spatially restricted (Fig. 3k, l). Altogether, these results demonstrate a crucial role of WNT and TGF-β signaling in the induction and level of *T/Bra* expression in *EpiTS* embryoids between 72 and 120 h. In contrast, the BMP pathway was shown to have an effect on the overall growth of embryoids, with a smaller effect on *T/Bra* expression between 96 and 120 h.

### Spontaneous axial morphogenesis of *EpiTS* embryoids

Previous embryoid models involving ESCs and TSCs in ETS embryos^11^ did not recapitulate the axial patterning of the early embryo. In our hands, ETS embryos could be generated at frequency (corresponding to multicellular aggregates comprising an embryonic and extraembryonic compartment) of 6.07% (*n* = 34/560) (Supplementary Fig. 6a). The ETS embryos expressed epiblast markers *Oct4* and *Otx2* at 120 h (Supplementary Fig. 6b). To test whether post-gastrulation developmental stages could be achieved in ETS embryos, we adopted our *EpiTS* embryoid culture approach by placing individual ETS embryos in U-bottom 96-wells. *T/Bra* expression was found to be highly variable, with some tissues showing a dispersed and others a more localized expression by 120 h (Supplementary Fig. 6c). When cultured until 240 h, ETS embryos with small embryonic-to-extraembryonic ratio were overgrown by TSC aggregates, and ETS embryos that were small in size (<200 µm) did not grow further. Interestingly, we observed that ETS embryos that upregulated *T/Bra* and had a larger embryonic compartment occasionally underwent axial elongation, suggesting that when grown in ECM- and serum-free culture conditions, these embryoids might reach post-gastrulation stages (Supplementary Fig. 6d).

**Fig. 4:**
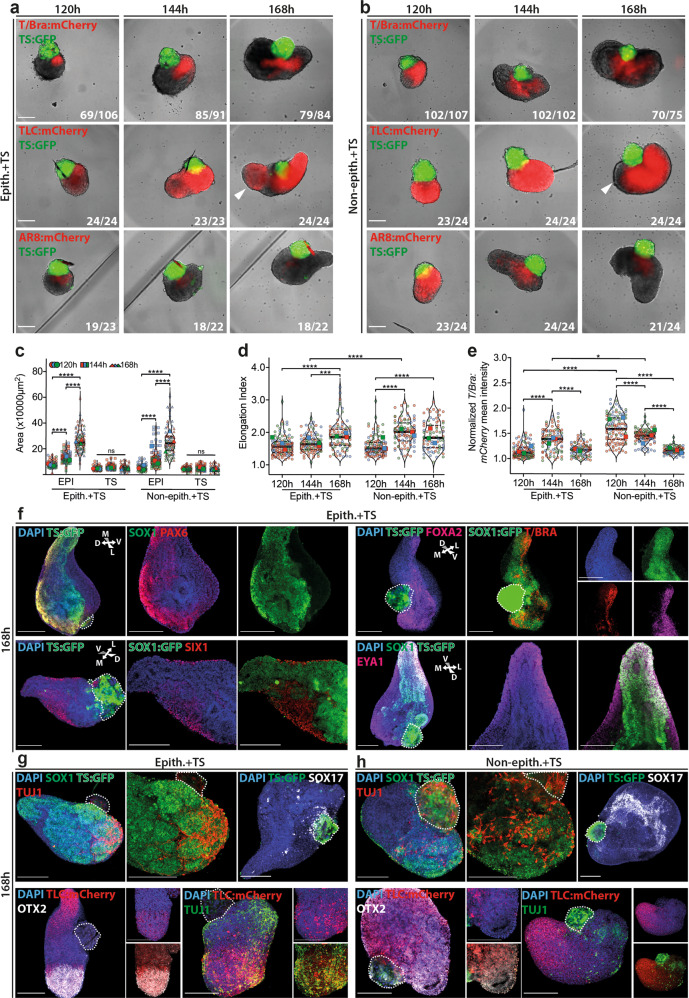
Axial morphogenesis in EpiTS embryoids. **a**, **b** Representative images showing *T/Bra:*mCherry (top), *TLC:*mCherry (middle), and *AR8:*mCherry (bottom) expression dynamics in epithelialized (**a**) and non-epithelialized (**b**) *EpiTS* embryoid between 120 and 168 h. **c**–**e** Quantification of total EPI vs TS area (**c**), elongation index (**d**), and background normalized *T/Bra:*mCherry mean intensity (**e**) in epithelialized or non-epithelialized embryoids between 120 and 168 h. For elongation index quantification, TS subtraction was performed. Data was collected from three biologically independent experiments. For **d**, adjusted *p*-values are: Epith.+TS (144 h) vs Epith.+TS (168 h), *p* = 0.0002. For **e**, Epith.+TS (144 h) vs Non-epith.+TS (144 h), *p* = 0.0277. **f** Representative confocal images showing *Sox1, Pax6, T/Bra, Foxa2* (top panel) *Six1, Eya1* (bottom panel) immunostainings at 168 h in epithelialized embryoids. **g**, **h** Representative confocal images showing *Sox1, Tuj1, Sox17* (top panel), and *mCherry, Otx2, Tuj1* (bottom panel) immunostainings at 168 h in epithelialized (**g**) or non-epithelialized (**h**) embryoids. Nuclei were stained with DAPI. TS cells were depicted with dashed line. All embryoids were formed from 100ESC/100TSC condition. For all conditions in **a**–**e**, number of mCherry-positive embryoids over total number of embryoids analyzed are indicated at bottom right of **a**, **b**. Large symbols indicate mean values of each replicate. Black lines indicate median and quartiles. For all statistical analysis, one-way ANOVA followed by Tukey multiple comparison test was performed. Following *P* value style was used: (****) < 0.0001, (***) 0.0002, (**) 0.0021, (*) 0.0332, (ns) 0.1234. Scale bars: 200 µm. Source data are provided as a Source Data file.

When 120 h old *EpiTS* embryoids were cultured in N2B27 medium with no additional growth factors, the embryonic compartment began to elongate (Fig. 4a, b). While epithelialized EPI aggregates alone remained spherical, non-epithelialized ones elongated efficiently^40^ (Supplementary Fig. 7a). The EPI compartment in *EpiTS* embryoids grew significantly over time, while the size of the TSC aggregates did not change markedly (Fig. 4c), suggesting that growth of extraembryonic compartment is not required for the elongation of the EPI domain. To quantify the dynamics of elongation, we performed image analysis to determine the axial length and elongation index of the embryoids (Supplementary Fig. 7b). This analysis revealed that in both epithelialized and non-epithelialized embryoids, the axial length and the elongation index significantly increased from 120 to 168 h (Fig. 4d, Supplementary Fig. 7c). *EpiTS* embryoids formed from non-epithelialized (or smaller) EPI aggregates were significantly more elongated at 144 h (Fig. 4d, Supplementary Fig. 7d, e), suggesting a role for the size and epithelial architecture of the starting EPI aggregate on the elongation dynamics (Supplementary Movies 5,6).

In embryoids generated from epithelialized tissues, *T/Bra* expression peaked around 144 h and gradually declined until 168 h, when it became restricted to the elongating tip and then trailed behind in a short stripe of cells. In contrast, non-epithelialized embryoids showed a constant decrease in the intensity and size of the *T/Bra* domain with increasing elongation (Fig. 4e, Supplementary Fig. 7f). WNT signaling was active in almost all embryoids and marked the *T/Bra-*expressing tip with no significant change in activity levels until 168 h (Supplementary Fig. 7g, h). In epithelialized embryoids, WNT activity reappeared at the anterior domain (Fig. 4a, b, middle panels, white arrows), resembling the TCF/LEF reporter activity in the tail and midbrain/hindbrain of E8.5 embryos^41^. Overall, TGF-β pathway activity was found to be lower and in close proximity to the extraembryonic compartment, excluded from the elongating tip (Fig. 4a, b, bottom panels; Supplementary Fig. 7i, j).

### Signaling and axial patterning in *EpiTS* embryoids

To better understand the underlying mechanism of axial morphogenesis in our embryoids, we investigated the roles of the WNT, TGF-β, and BMP pathways by inhibiting their activity between 96 and 120 h. In general, treatment with inhibitors resulted in significant size reduction by 168 h (Supplementary Fig. 8a). Dkk1 and Lefty led to a reduction of *T/Bra-*positive embryoids, compared to Noggin. Embryoids treated with Lefty exhibited small clumps of *T/Bra*-positive cells, while Dkk-treated ones showed a more scattered expression throughout the body of the structure (Supplementary Fig. 8b). In Noggin-treated embryoids, *T/Bra* expression was retained within a larger domain compared to untreated or Dkk1/Lefty-treated ones (Supplementary Fig. 8c). Notably, the elongation frequency was found to be significantly higher in the presence of Noggin (Supplementary Fig. 8d, Supplementary Movies 7–10). It is conceivable that excessive BMP signaling from the extraembryonic ectoderm could impair axial elongation, and treatment with Noggin could mitigate this effect, consistent with the antagonistic role of BMP4 on axial elongation of gastruloids^42^. In these embryoids, *T/Bra*-positive cells were often organized into longer stripes that co-expressed *FoxA2*, suggestive of a more developed notochord or an anterior mesendoderm (AME)-like structure (Supplementary Fig. 8e). In support of this, we detected higher expression levels of *T/Bra*, *Shh*, *FoxA2*, and *Gsc* with Noggin-treated compared to untreated embryoids (Supplementary Fig. 8f), in line with previous reports defining a role for Noggin in proper notochord formation^43^.

At 168 h, epithelialized embryoids acquired an expanded neural identity, as evidenced by *Sox1* expression spanning from the elongated tip towards the opposite end. Interestingly, we could detect polarized expression of *Pax6* and *T-Bra/Foxa2*, all partially overlapping with *Sox1* expression, suggesting a dorsal–ventral organization similar to the developing neural tube^44^. Furthermore, the neural tissue was flanked by *Six1*+ *Eya1*+ paraxial mesoderm derivatives (Fig. 4f), suggesting an organization across a medio-lateral axis. We detected numerous *Tuj1*-positive neurons within *Sox1*-positive rosettes located on the opposite end of the elongated tip. The rosettes and neurons showed high WNT activity colocalized with *Otx2* expression, indicating establishment of an anterior–posterior axis to include a ‘brain-like’ tissue. Moreover, only few *Sox17*-positive cells were found scattered in the vicinity of the extraembryonic domain, suggesting poor endoderm differentiation (Fig. 4g). In contrast, non-epithelialized embryoids showed a smaller domain of *Sox1* expression comprising comparable numbers of *Tuj1*-positive neurons with relatively short axonal projections. Compared to epithelialized embryoids, the *Otx2* expression domain was more restricted and showed low levels of WNT activity. *Tuj1*-positive neurons were also located away from the WNT-positive elongated tip. Interestingly, the expression domain of *Sox17* in these embryoids was much larger and located at the anterior end of the embryoids colocalizing with *Foxa2* (Fig. 4h, Supplementary Fig. 8g), similar to endoderm tissue seen in gastruloids^6^. Collectively, these data demonstrate the capacity of *EpiTS* embryoids to spontaneously undergo axial morphogenesis to establish multi-axial patterning. Importantly, the presence or absence of an epithelium in the starting EPI aggregate directly influences the elongation dynamics as well as the proportions of the tissue types that are generated.

### Single-cell RNA-sequencing reveals distinct differentiation trajectories in *EpiTS* embryoids

**Fig. 5:**
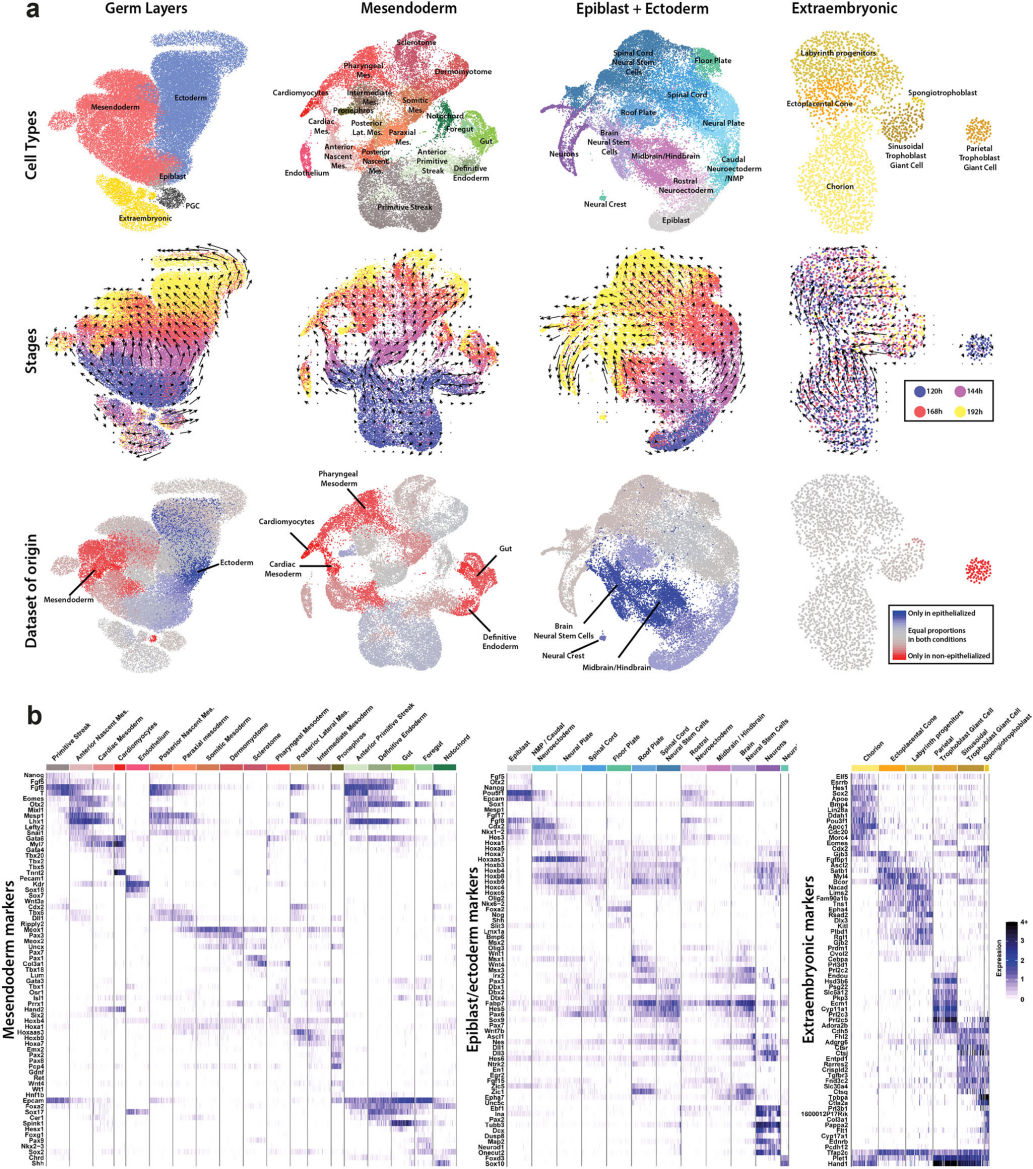
Comparative single-cell RNA-sequencing analysis of EpiTS embryoids. **a** UMAP plots showing cell types, RNA velocity overlayed stages, and datasets of origins in germ layers, mesendoderm, epiblast + ectoderm, and extraembryonic clusters. **b** Heatmap plot of selected marker genes from mesendoderm, epiblast + ectoderm, and extraembryonic clusters. PGC primordial germ cells, Lat. lateral, Mes. mesoderm.

To shed light on the cell-type composition and diversity of *EpiTS* embryoids, we performed single-cell RNA-sequencing (scRNA-seq) analysis between 120 and 192 h (Fig. 5). We could identify that from 120 h onwards, cells mapping to an epiblast-state gradually differentiated into ectoderm or mesendoderm lineages (Fig. 5a, germ layers). Primordial germ cells and extraembryonic tissue had a distinct transcriptional identity that segregated away from the germ layers observed in our embryoids. RNA velocity on extraembryonic cells showed no clear temporal differentiation trajectory; however, we identified distinct cell populations that suggested a compartmentalization within the extraembryonic tissue (Fig. 5a, extraembryonic). At 120 h, the majority of the extraembryonic tissue had chorion identity (*Cdx2, Elf5, Eomes, Esrrb*) and expressed *Bmp4*, supporting involvement of BMP signaling for the initiation of gastrulation-like events in our embryoids. Interestingly, we detected *Furin* and *Pcsk6* expression that could suggest the processing of embryonic Nodal by the extraembryonic tissue^45^. We further identified clusters of cells that map to ectoplacental cone (*Fgfbp1, Ascl2*) or labyrinth progenitors (*Gjb2, Dlx3, Ovol2*) that demonstrate multipotentiality of chorionic ectoderm. At later stages of culture, we detected trophoblast giant cells (*Hand1, Ctsq, Prl2c2*) and a few spongiotrophoblast cells (*Tpbpa, Flt1*) (Fig. 5b, extraembryonic markers, Supplementary Fig. 9a–d), in line with previous in vitro TSC differentiation data^46^.

A closeup on the mesendoderm cluster revealed complex differentiation trajectories (Fig. 5a, mesendoderm). At 120 h, the majority of cells had a primitive streak identity (*T, Mixl1, Wnt3*), that progressively branched to give rise to endoderm (*Sox17, Spink1*) and notochord (*Noto, T, Shh*) tissues via an anterior primitive streak-like population (*Foxa2, Gsc*). Cells mapping to the nascent mesoderm (*Mesp1, Hoxb1*) diverged into two distinct trajectories: the anterior portion gave rise to cardiac mesoderm (*Myl7, Gata4, Hand2*) and cardiomyocytes (*Ttn, Nkx2-5, Mef2c*)^7^, while the posterior nascent mesoderm cells differentiated towards paraxial mesoderm (*Msgn1, Tbx6, Meox1*). The paraxial mesoderm further diverged into somitic (*Meox2, Pax3, Uncx*), posterior lateral mesoderm (*Msx2, Foxf1, Hoxc6*) or intermediate mesoderm (*Osr1, Gata3, Wt1)* tissues. From 168 to 192 h of culture, the abovementioned mesoderm populations gave rise to more differentiated pharyngeal mesoderm (*Prrx1, Isl1, Tbx1*), sclerotome (*Pax1*), and dermomyotome (*Dmrt2*) cells (Fig. 5b, mesendoderm markers, Supplementary Fig. 10a, b).

A detailed RNA-velocity analysis of the ectoderm cluster revealed that between 120 and 144 h, epiblast cells (*Oct4, Nodal*) gave rise to spinal cord tissue *(Sox1, Pax6, Hoxb7/8/9*) (Fig. 5a, epiblast + ectoderm). We noted a striking compartmentalization among the cells of the spinal cord, with differentiation towards dorsal (*Wnt1, Dbx2, Pax7*) and ventral (*Olig2, Foxa2, Shh*) cells of the neural tube. On a second trajectory, epiblast cells differentiated to generate midbrain/hindbrain tissue. The lower expression level of midbrain markers (*En1/2, Pax5)* and higher expression level of hindbrain markers (*Egr2, Irx2, Epha7, Fst)* could suggest that the ‘brain-like’ tissue generated in the embryoids was primarily of hindbrain origin. In support of this, we detected *Hoxa2, Hoxb1/2*, and *Cyp26* expression in the hindbrain-like tissue, while more posterior *Hoxa3/b3* and *Hoxb4* were excluded from this region (Fig. 5b, epiblast + ectoderm markers; Supplementary Fig. 11a, b), suggesting that the hindbrain tissue in our embryoids corresponds to rhombomeres 1–6^47^. At later stages, we detected neural stem cells (*Pou3f3, Ascl1, Nes*) that either had midbrain/hindbrain (*Zic1/5, Msx3*) or spinal cord (*Hoxa4/b4*) identities. These neural stem cells generated dorsal interneurons (*Lhx1/5, Pou4f1*) and ventral motor neurons (*Isl1/2, Nkx6-1 Phox2a/b*) at both spinal cord (*Hoxb7/8/9*) and hindbrain (*Lhx2/9, Barhl1/2*) levels (Supplementary Fig. 12a–d), revealing a remarkable recapitulation of the cell-type diversity similar to the in vivo situation^48^.

We then used the scRNA-seq dataset to systematically assess the effect of the initial epithelial architecture of embryoids on the resulting cell-type diversity. A comparative analysis of epithelialized and non-epithelialized embryoids showed that several tissue types, including cardiac mesoderm, definitive endoderm, and pharyngeal mesoderm derivatives, were almost exclusively found in non-epithelialized embryoids (Fig. 5e, Supplementary Fig. 13a, b). Moreover, the neural tissues in non-epithelialized embryoids were shown to be composed of spinal cord tissue. Conversely, midbrain/hindbrain tissue, brain-specific neural stem cells, and their respective neurons were only detected in epithelialized embryoids (Supplementary Fig. 12e). These results demonstrate that the epithelial architecture of initial EPI aggregates has a direct impact on embryonic developmental trajectories and promotes neural differentiation in embryoids.

## Discussion

We have developed a bioengineered embryoid culture system for the study of early mouse post-implantation development that combines aggregates of ESCs and TSCs. Unlike previous studies^11,12,49^, this culture method allows simple independent modulation of the embryonic and extraembryonic compartments and makes our system both experimentally tractable and efficient for quantitative and mechanistic studies.

The controlled interaction between EPI and TSC aggregates leads to *EpiTS* embryoids that resemble the early mouse embryo. Specifically, EPI aggregates that have an apicobasally polarized epithelium develop a polarity with high *T/Bra* and low *Sox2/Otx2* expression on one end, and no *T/Bra* and high *Sox2/Otx2* expression on the rest of the aggregate. Furthermore, at this time the lumen of the epithelium collapses and while the *T/Bra*-expressing cells appear to undergo an EMT, cells on the opposite side remain organized in epithelial rosettes. This organization resembles the anterior–posterior axis of the E6.5 embryo and strictly depends on the initial size of the EPI aggregate. We identify BMP signaling from the extraembryonic compartment, via WNT/TGF-β pathways, as the key regulator of polarized *T/Bra* expression in *EpiTS* embryoids.

A significant finding of our study is the effect that an epithelium has on *EpiTS* embryoid development. Under the same culture conditions, *T/Bra* expression is much more restricted in epithelialized than in non-epithelialized EPI aggregates. This suggest that the formation of an epithelium raises the threshold for WNT and TGF-β signal response such that, after the onset of *T/Bra* expression at one end, the probability that a second event happens in the aggregate end is low and this contributes to the restricted patterning of the EPI. The culture conditions we use here sensitize the EPI compartment to *T/Bra* expression which, in non-epithelialized aggregates, initially spreads throughout the aggregate. However, in the original gastruloid protocol, ESC aggregates undergo spontaneous symmetry breaking in the absence of any extraembryonic signals, localized to one end of the aggregate. Altogether, these observations suggest that one role of the extraembryonic ectoderm is to bias the spontaneous symmetry breaking event and that the epithelial organization of the EPI contributes to this by raising the threshold for signal response. The mechanism for this is unclear at the moment but the junctional organization of signaling receptors in the epiblast^50,51^ is likely to play a role. The situation we observe in *EpiTS* embryoid phenocopies aspects of the activity of the AVE where BMP, TGF-β, and WNT signaling inhibitors secreted from the Anterior Visceral Endoderm (AVE) inhibit the spread of *T/Bra* expression, contributing to the anterior–posterior patterning of the embryo, suggesting that the epithelial organization of the epiblast contributes to this effect.

Long-term culture of *EpiTS* embryoid*s* in growth factor-free medium resulted in axial elongation (originating from the *T/Bra*-expressing pole) and subsequent multi-axial patterning. The extent of this process was dependent on the proportions of cell types generated as a function of the initial tissue architecture of the EPI aggregates. For example, we detected cardiac mesoderm and definitive endoderm derivatives almost exclusively in non-epithelialized embryoids, resembling post-occipital patterning in *gastruloids*^6^. In both cases the EPI compartment displayed expanded WNT and TGF-β signaling at early stages of culture, and this is likely to cause the observed loss of anterior neural progenitors. In contrast, epithelialized embryoids, although also exhibiting *T/Bra* expression initially, later are depleted for mesodermal derivatives and display cell fates associated with midbrain/hindbrain and, in some instances, spinal cord levels, supporting the importance of an epithelium for the formation of brain tissues^52,53^. The organization of epithelialized embryoids can be construed as an ‘early embryo’ in which the primitive streak is initiated but immediately truncated, as they lacked most of the mesendodermal derivatives. This could suggest that neural induction has taken place in these embryoids. In support of this, late *EpiTS* embryoids had *T/Bra*+*Foxa2*+ tissue extending along the embryoids suggesting the existence of a notochord/anterior mesendoderm (AME)-like structure which in the embryo is responsible for the induction of the midbrain/hindbrain^54^.

A surprising feature of the emergence of brain structures in the *EpiTS* embryoids is that it occurs in the absence of an AVE which, in the embryo, is thought to be needed to preserve neural potential in the epiblast^23^. An earlier study has shown that gastruloids derived from a combination of XEN cells and ESCs could trigger an epithelium^12^ and develop cerebral cortex-like tissue^55^. In the embryoids that lack extraembryonic endoderm cells, we do not observe any forebrain tissue, supporting this possibility. This could potentially be overcome by WNT inhibition at later stages of the culture, since Dkk1 secreted from the prechordal plate is known to be instructive for forebrain formation^56^. Together with our results, these observations suggest that the AVE might be involved in maintaining an intact epithelium in the anterior region of the epiblast^57^, and that this might be sufficient to preserve the neural potential, which is later developed by the AME during neural induction.

Our experiments show how bioengineering-inspired approaches can be exploited to standardize complex embryoid models and allow for the modeling of embryonic-extraembryonic cell/tissue interactions involved in the generation of precise patterns during development. *EpiTS* embryoids should serve as a canvas to generate developmentally relevant organ-specific structures of either neural or mesendoderm origin, through an in-vivo-like gastrulation process. Traditional approaches to identify tissue origins in the embryo have relied largely on genetic manipulations which are labor intensive. *EpiTS* embryoids thus provide an alternative approach to decipher the roles of mechanics and gene expression, which is difficult to perform in vivo. Ultimately, the adoption of our approach to generate human embryoids holds significant potential to shed light on early human development as experimenting with early human embryos is challenging.

## Methods

### Cell culture

Mouse embryonic stem cells (SBr line^58^) were cultured at 37 °C in 5% CO_2_ in medium composed of DMEM + Glutamax (#61965-026), 10% ES cell-qualified FBS (#16141-079), 1 mM sodium pyruvate (#11360-070), 1× MEM nonessential aminoacids (#11140-035), 0.1 mM 2-mercaptoethanol (#31350-010), and 1000 u/ml Pen/Strep (#15140-122) supplemented with 3 µm GSK3i (#361559), 1 µm MEKi (#S1036), and 0.1 µg/ml LIF (in house preparation). Cells were routinely passaged every 2–3 days by seeding 8000–9000 cells/cm^2^ and every 20 passages a fresh vial was thawed. Cells were tested and confirmed free of mycoplasma. Mouse trophoblast stem cells (TS:GFP line^59^) were cultured at 37 °C in 5% CO_2_ in TS medium composed of RPMI 1640 + Glutamax (#61870-010), 20% ES cell-qualified FBS (#16141-079), 1 mM sodium pyruvate (#11360-070), 0.1 mM 2-mercaptoethanol (#31350-010), and 1000 u/ml Pen/Strep (#15140-122). TS medium was conditioned on irradiated MEFs for 3 days and stored at −20 °C. This was repeated three times for one batch of irradiated MEFs. Aliquots of TS conditioned medium (TSCM) were thawed and mixed 3:1 with fresh TS medium before cell passaging. 50 ng/ml Fgf4 (#100-31) and 1 µg/ml Heparin (#H3149) were added to make final TS medium. TS cells were routinely passaged every 2–3 days by seeding 5000–6000 cells/cm^2^ and every 20 passages a fresh vial was thawed. MEFs were prepared in house and stocks were prepared at passage 1. They were cultured in TS medium with no additional growth factors. For the co-culture experiments, MEFs from passages 4–7 were used. Cells were tested and confirmed free of mycoplasma.

### Preparing EPI and TS differentiation medium

N2B27 medium was prepared by 1:1 mixing of DMEM/F12+Glutamax (#31331-028) and Neurobasal (#21103-049) with the addition of 0.5× N2 supplement (#17502001), 0.5× B27 supplement (#17504001), 0.5× Glutamax (#35050-038), 1 mM sodium pyruvate (#11360-070), 1× MEM nonessential aminocacids (#11140-035), 0.1 mM 2-mercaptoethanol (#31350-010), and 1000 u/ml Pen/Strep (#15140-122). 12 ng/ml Fgf2 (#PMG0035), 20 ng/ml Activin-A (#338-AC), and 1% KSR (#10828-010) were added to make final EPI differentiation medium (EPIdiff). TS differentiation medium (TSdiff) was prepared by 1:1 mixing of N2B27 and TS medium supplemented with 25 ng/ml Fgf4 (#100-31) and 500 ng/ml Heparin (#H3149).

### Preparing EPI and TSC aggregates on PEG microwells

Poly(ethylene glycol) (PEG) microwells with 400 µm well diameter (121 wells per array) were prepared on 24-well plates as previously descrbed^13^. Microwells were equilibrated with 50 µl of either EPIdiff (for ES cells) or TSdiff (for TS cells) for at least 30 min at 37 °C. Mouse ES and TS cells and were dissociated to single cells with Accutase (#A11105-01) or TrypLE (#12605-028), respectively. Cells were then centrifuged at 112 × *g* for 5 min and washed twice with 10 ml PBS at 4 °C. Cells were resuspended in cold EPIdiff (for ES cells) or TSdiff (for TS cells) and suspension of 484.000 cells/ml was prepared. 35 µl of the suspension was added dropwise on microwell arrays to have 100–150 cells/well. Seeding was done at 37 °C for 15 min. Growth factor reduced Matrigel (#356231) was diluted in cold EPIdiff or TSdiff to 3% or 2% (v/v), respectively. The medium was vortexed and 1 ml was slowly added from the side of the well, avoiding direct addition from the top of the microwell arrays. Plates were kept at 37 °C in 5% CO_2_ for at least 72 h before further processing.

### Forming *EpiTS* embryoids

At 72–75 h of culture, aggregates on microwell arrays were flushed out and transferred to non-tissue culture treated 10 cm plates in 10 ml warm N2B27 medium. Single EPI and TSC aggregates were picked in 10 µl and transferred to low adherent U-bottom 96-well plates (#COR-7007). 170 µl of N2B27 medium was added on top. At 96 and 120 h, 150 µl of medium was replaced with fresh N2B27 and *EpiTS* embryoids were generally kept until 168 h. Protein inhibitors Lefty (#746-LF-025), Noggin (in house preparation), Dkk1 (#5897-DK-010) were added at indicated timepoints at 200 ng/ml final concentration. Small molecule inhibitors; SB431542 (#S4317), XAV939 (#S1180), LDN193189 (#SML0559) were added at indicated timepoints at 10 µm, 10 µm, and 1 µm final concentrations, respectively.

### Forming ETS embryos

ETS embryos were generated as previously described^11^. Briefly, mESCs were dissociated into single cells by accutase and washed with PBS. In parallel, TSCs were dissociated with trypsin into small clumps of 4–5 cells and washed with PBS. Five thousands mESCs and 5000 TSC clumps were counted and mixed in total of 120 µl Matrigel. From the suspension, 15 µl drops were casted onto µ-Slide 8-well plates. After gelation, 400 µl of TSdiff medium was added and plates were kept at 37 °C in 5% CO_2_ for 24 h. Next day and on third day medium was changed with 400 µl fresh TSdiff.

### Testing effect of cytoskeletal inhibitors on EPI aggregate epithelialization

EPI aggregates were formed in the presence of Matrigel with following inhibitors until 72 h: 1 µm LPA(#L7260), 10 µm Y27632 (#72302), 10 µm Blebbistatin (#72402), 1 µm ML-7 (#4310), 5 mm Calyculin-A (#9902 S), 100 nm Cytochalasin-D (#250255), 1 µm Latrunculin-A (#L5163), 100 nm Jasplakinolide (#J4580), 50 µm CK-666 (#SML0006), 25 µm BpV (#S8651).

### Culturing of EPI aggregates with protein coated beads or on transwells

For bead experiments, Cytodex 3 microcarriers (#17048501) were resuspended in HBSS (Ca^++^, Mg^++^) at 40,000 beads/ml concentration. Six hundred beads were transferred in 0.5 ml eppendorfs and Fgf2 (#PMG0035), Activin-A (#338-AC), Wnt3a (in house preparation), or Bmp4 (#120-05ET-100) was added at 25 µg/ml final concentration. Beads were incubated at 4 °C overnight. Next day, beads were washed repeatedly to reach 1:250,000 dilution in N2B27 and transferred in 10 µl to low adherent U-bottom 96-well plates together with epithelialized EPI aggregates.

For transwell experiments, EPI aggregates were placed in 190 µl to low adherent U-bottom 96-well plates and 1, 3, or 5 TSC aggregates were placed in transwells on top (#CLS3374). Forty microliters of medium was added to cover TSC aggregates. At 96 h, 180 µl of medium was replaced with fresh N2B27.

### Immunostaining and confocal microscopy

*EpiTS* embryoids were washed with PBS and fixed with 4% PFA for 2 h at 4 °C. PFA was removed by three serial washes of 20 min at room temperature. Blocking was performed in blocking solution (PBS + 10%FBS + 0.3% Triton-X) for 1 h at room temperature. Primary antibodies (see Supplementary Table 1) were incubated for at least 24 h at 4 °C in blocking solution. Next day, primary antibodies were removed by three serial washes of 20 min at room temperature. Secondary antibodies were incubated for 24 h and next day embryoids were washed and mounted on glass slides in mounting medium. Confocal images were taken using an LSM700 inverted (ZEN software, Zeiss) with EC Plan-Neofluar ×10/0.30 or Plan-Apochromat ×20/0.80 air objectives.

### Image analysis

All images were processed using algorithms developed in Image J (version 2.0.0-rc-69/1.52n). Brightfield, GFP (for TS cells), and mCherry (for *T/Bra)* channels were used as input. Thresholding and segmentation was performed sequentially for each channel. An embryoid was considered positive if the signal for the reporter was at least 30% more than the background and had an area of at least 1500 µm^2^. Area of EPI domain was calculated by subtraction of the area of the object identified in GFP channel from the area of the object identified in brightfield channel. For the calculation of the anterior EPI domain, area of the object identified in mCherry channel was subtracted from the EPI area. *T/*Bra coverage index was calculated by dividing area of the object identified in mCherry channel to the EPI area. For morphology measurements, brightfield images were thresholded and segmented. Maximum inscribed circle function was used to fit circles in the identified object. Axial length was determined by connecting centers of the fit circles. Elongation index was calculated by dividing axial length to the diameter of the maximum inscribed circle. All image analysis codes are available upon request. Data was analyzed by using Microsoft Excel and GraphPad Prism.

### RNA isolation and qPCR

RNA was extracted with the RNeasy Micro kit (QIAGEN), according to manufacturer’s instructions and quantified with a spectrophotometer. One microgram of RNA was reverse transcribed with the iScript cDNA Supermix (#1708890). cDNA was diluted 1:5 and amplified by using Power SYBR Green PCR Master Mix. qPCR was run with a 7900HT Fast PCR machine (#4329001), using Power SYBR Green PCR Master Mix (Applied Biosystems), with an annealing temperature of 60 °C. Gene expression was normalized on Gapdh expression. Relative fold expression was calculated with the 2−ΔΔCT method. Primers used are indicated in the Supplementary Table 2.

### Bulk RNA-seq

EPI and TSC aggregates (formed from 100 cells/well condition) were lysed with 200 µl trizol, followed by addition of 70 µl of chloroform to trigger phase separation and then the aqueous phase was collected. The extraction process was repeated a second time, and an equal volume of isopropanol was added to precipitate the RNA, which was collected by centrifugation at 20,000 × *g* for 30 min. The pellet was washed with 15 mM sodium acetate in aqueous 70% ethanol, followed by salt-free 70% ethanol, before picking up in RNAse free water. RNA quantity and quality were assessed on nanodrop, qubit, and Agilent TapeStation 4200 profiling, and showed absorbance ratios 260/280 of 1.85 ± 0.12 and RNA integrity numbers (RIN) of 9.9 ± 0.2 (average ± SD), supporting good purity and absence of degradation. TruSeq stranded mRNA LT libraries were prepared according to Illumina protocol 15031047 Rev. E, starting from 300 ng of RNA, quantified by qubit DNA HS, profiled on TapeStation 4200, and sequenced on an Illumina HiSeq 4000 at a targeted depth of 36 Mreads/sample and paired-end read length of 81,8i,8i,81. The reads were trimmed for adapters with bcl2fastq v2.20.0, aligned to the mouse genome mm10 with STAR 2.7.0e, and a count matrix was assembled using the cellranger v4.0 curation of ENSEMBL annotations. In the manuscript, “Gene expression” refers to natural logarithm of counts per million for bulk RNA-seq data. The data were collected from four independent experiments.

### Single-cell RNA-seq

Single-cell RNA-seq of *EpiTS* embryoids (formed from 100ESC/100TSC condition) was performed with 10× single-cell 3’ gene expression reagent kit chemistry v3.1, according to the protocol CG000204 Rev D. Library QC was performed on an Agilent TapeStation 4200, and sequencing was performed on Illumina HiSeq 4000, with a paired-end read length of 28, 8i, 110, targeting ~3000 cells and 300 Mreads per library (which included two replicates per condition, 4 timepoints, epithelialized and non-epithelialized embryoids, for a total of 16 libraries). Recovered cells were 3300 ± 900, and reads per cell after UMI collapse of 29,000 ± 4500 (average ± SD). The reads were trimmed for adapters and polyA with cutadapt^60^ v2.1, and aligned to mm10 with cellranger 3.1. The count matrices were imported in R v3.6 with Seurat^61^ v3.1. Live cells were selected based on detection of more than 2000 genes, and mitochondrial gene content of 1.5–15%, and counts were normalized as natural logarithm of counts normalized to 10k per cell (referred to as “Gene expression” in the manuscript). A mini-analysis including dimensionality reduction by PCA based on centered and scaled most variable genes and cell-type annotation transfer from an in vivo atlas^62^ with scmap^63^ was performed on individual datasets for the needs of doublet removal with DoubletFinder v2.0.3^64^. After data filtering, all datasets were merged, variable genes, scaling and PCA repeated, and batch correction was then done with Harmony v1^65^. A first Louvain clustering yielded clusters that were assigned to germ layers. UMAPs^66^ were computed with uwot v0.1.5, first with 100 cells per cluster and spectral initialization, and then with all cells, initialized on the previous cluster centroids plus noise. The data were then split by germ layers, and a refined Louvain clustering and UMAP view was computed for each subset. Louvain clusters were finally assigned to cell types based on canonical markers. Custom code was used for visualizing cell-type proportions vs day and/or epithelialization, normalizing for heterogeneous dataset sizes to avoid bias. A further closeup was similarly performed on the neurons of the epiblast + ectoderm subset. Splicing data were estimated with velocyto^67^ v0.17, and RNA velocity was computed with scVelo v0.2.1^68^ in deterministic mode, using python v3.7.2 and scanpy v1.5^69^.

### Reporting summary

Further information on research design is available in the Nature Research Reporting Summary linked to this article.

## Supplementary information

Supplementary Information

Description of Additional Supplementary Files

Supplementary Movie 1

Supplementary Movie 2

Supplementary Movie 3

Supplementary Movie 4

Supplementary Movie 5

Supplementary Movie 6

Supplementary Movie 7

Supplementary Movie 8

Supplementary Movie 9

Supplementary Movie 10

Reporting Summary

## Data Availability

The single-cell RNA-sequencing data generated in this study have been deposited in the GEO database under accession code “GSE171209”. The bulk RNA-sequencing data used in this study are available on the GEO database under accession code “GSE171211”. All other relevant data supporting the key findings of this study are available within the article and its Supplementary Information files or from the corresponding author upon reasonable request. Source data are provided with this paper.

## Source data

Source Data

## Code availability

The codes for RNA-sequencing data exploration^70^ and for morphological feature quantification^71^ of embryoids can be accessed at Zenodo.
