# Supplementary material for: Bioengineered embryoids mimic post-implantation development in vitro
Source: Nat Commun. 2021 Aug 26;12:5140. doi: 10.1038/s41467-021-25237-8 (PMC8390504; doi:10.1038/s41467-021-25237-8)
Supplement: Supplementary file 1 — Supplementary Information [file 41467_2021_25237_MOESM1_ESM.pdf]

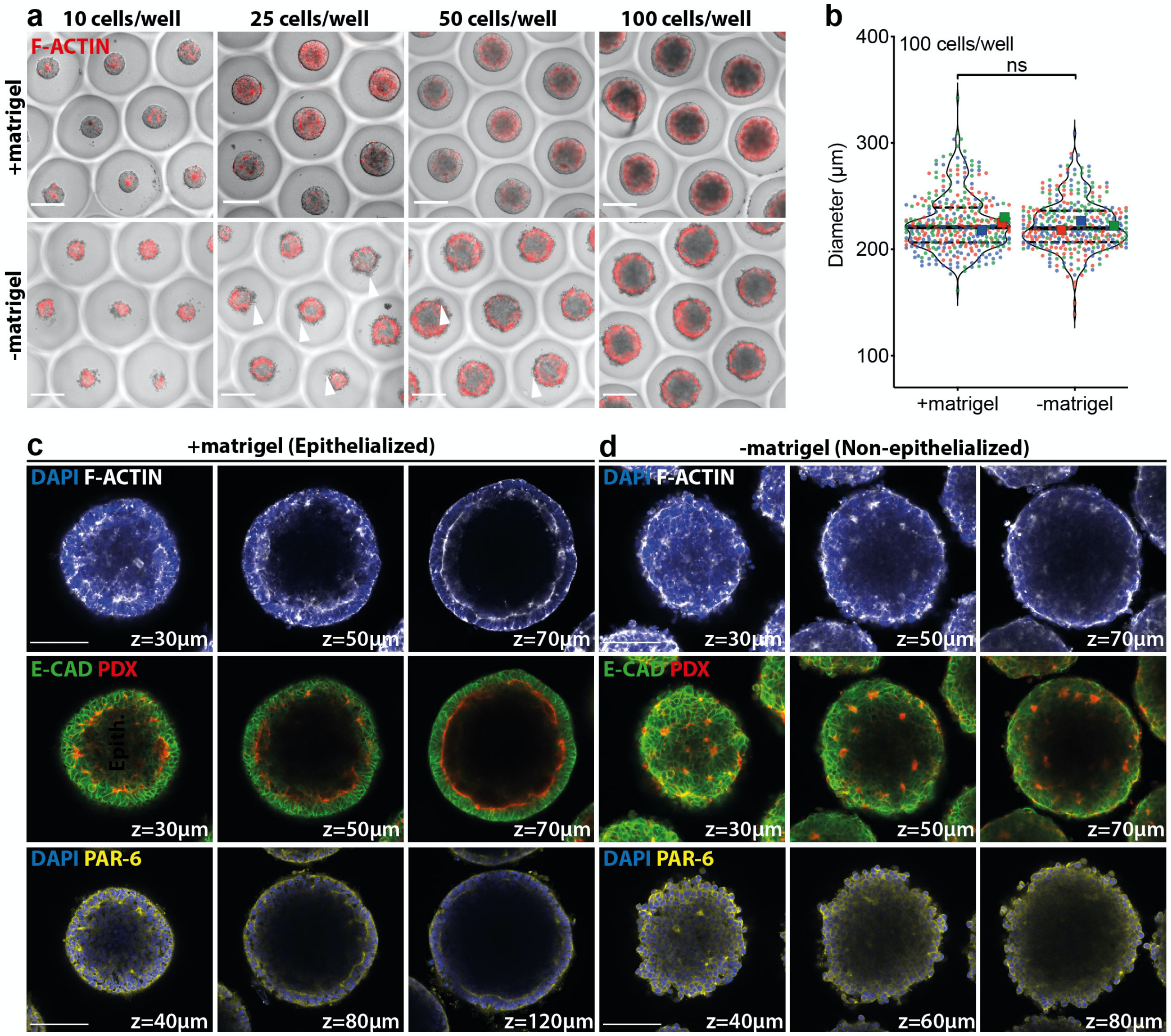

Supplementary Figure 1: Effects of starting cell number and addition of Matrigel on epithelialization of EPI aggregates. a) Representative confocal images at 72 h showing the effects of starting cell number and addition of Matrigel on *F-actin* expression (phalloidin staining). Note the shed cells around the aggregates (white arrows) in the absence of Matrigel. **b)** Comparing minimum ferret diameters of EPI aggregates at 72 h formed with or without Matrigel from 100 cells/well. For +matrigel and -matrigel conditions, total number of aggregates analyzed were 361 and 348, respectively. Data is collected from three biologically independent experiments. Large symbols indicate mean values of each replicate. Black lines indicate median and quartiles. **c,d)** Confocal images of showing multiple z-planes of EPI aggregates formed from 100 cells/well with **(c)** or without **(d)** Matrigel fixed at 72 h and stained for *F-actin* (phalloidin), *E-cadherin*, *Podocalxyin* and *Par6*. Nuclei were stained with DAPI. For statistical analysis, two-tailed unpaired Student's t-test **(b)** was performed. Following P-value style was used: (****)<0.0001, (***) 0.0002, (**) 0.0021, (*) 0.0332, (ns) 0.1234. Scale bars: 100µm. Source data are provided as a Source Data file.

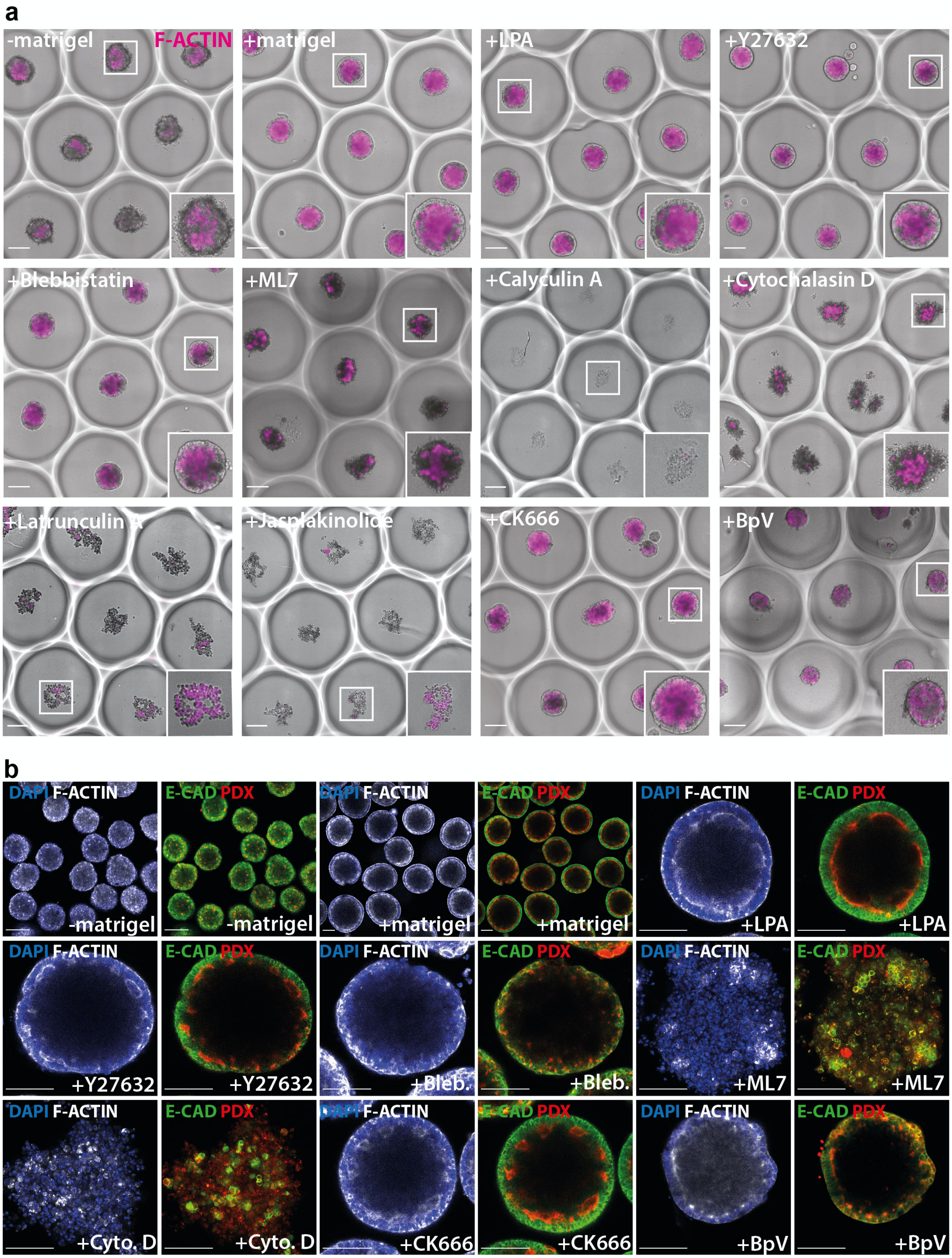
a
-matrigel
F-ACTIN
+matrigel
+LPA
+Y27632
+Blebbistatin
+ML7
+Calyculin A
+Cytochalasin D
+Latrunculin A
+Jasplakinolide
+CK666
+BpV
b
DAPI F-ACTIN
E-CAD PDX
-matrigel
+matrigel
+LPA
+Y27632
+Bleb.
+ML7
+Cyto. D
+CK666
+BpV

Supplementary Figure 2: Effect of cytoskeleton inhibitors on epithelialization of EPI aggregates. a) Representative images showing *F-actin* (phalloidin) expression in EPI aggregates on microwells, cultured with Matrigel and indicated inhibitors for 72 h. **b)** Representative confocal images of EPI aggregates cultured with Matrigel and indicated inhibitors for 72 h, showing *E-cadherin, Podocalyxin* and *F-actin* (phalloidin) expression. All EPI aggregates were formed from 100 cells/well. Scale bars: 100µm.

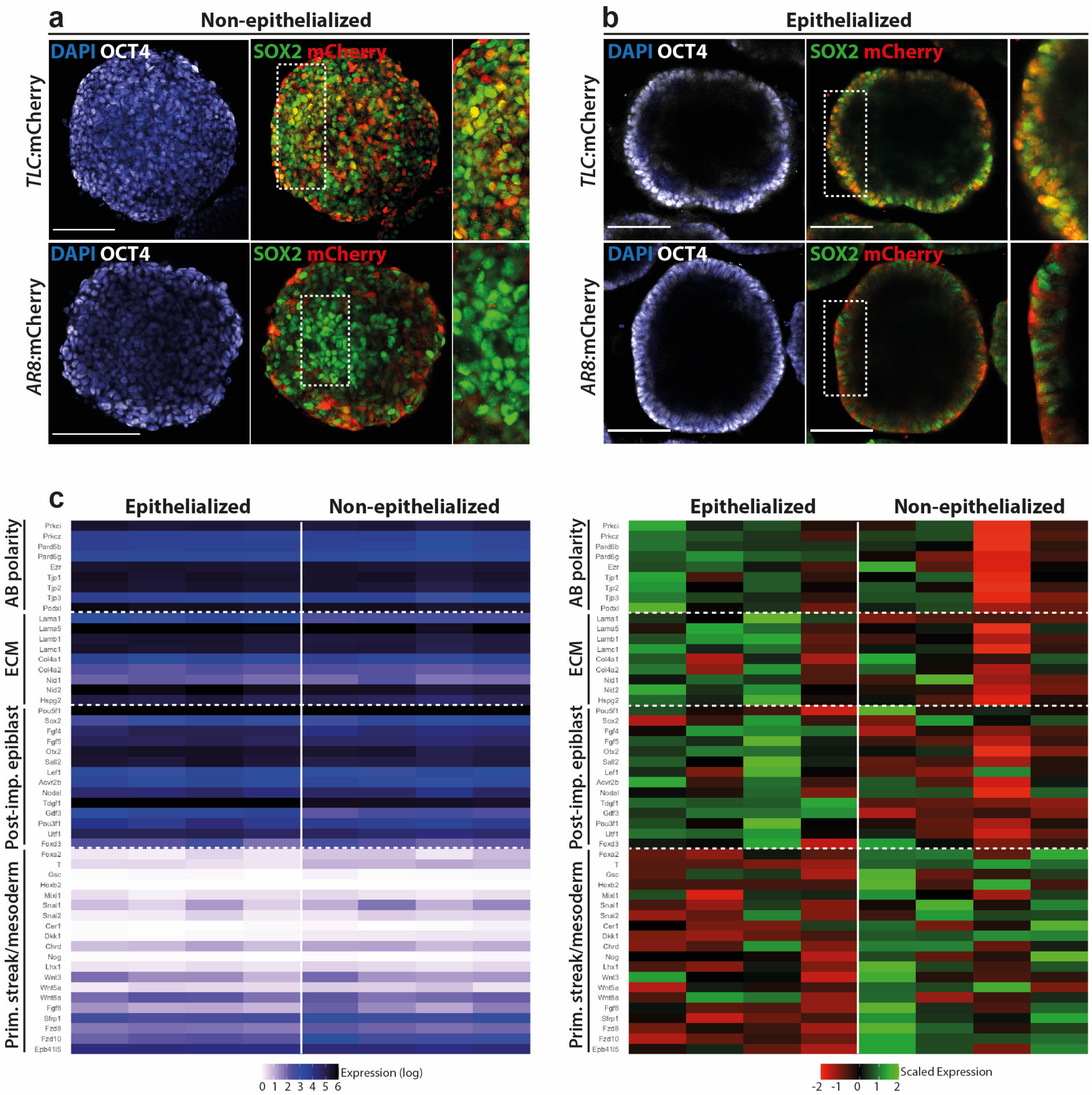

Supplementary Figure 3: WNT and TGF-βsignaling in EPI aggregates. a,b) Representative confocal images showing *Oct4, Sox2* expression and WNT *(TLC:mCherry*, top) or TGF-β (*AR8:mCherry*, bottom) reporter activity in non-epithelialized **(a)** or epithelialized **(b)** EPI aggregates at 72 h. All EPI aggregates were formed from 100 cells/well Scale bars: 100µm. **c)** Bulk RNA sequencing analysis of epithelialized and non-epithelialized EPI aggregates at 72 h formed from 100 cells/well, showing expression levels of apicobasal (AB) polarity, extracellular matrix (ECM), post-implantation epiblast and primitive streak/mesoderm genes. Scaled expression is showed in right panel. Data is collected from four biologically independent experiments.

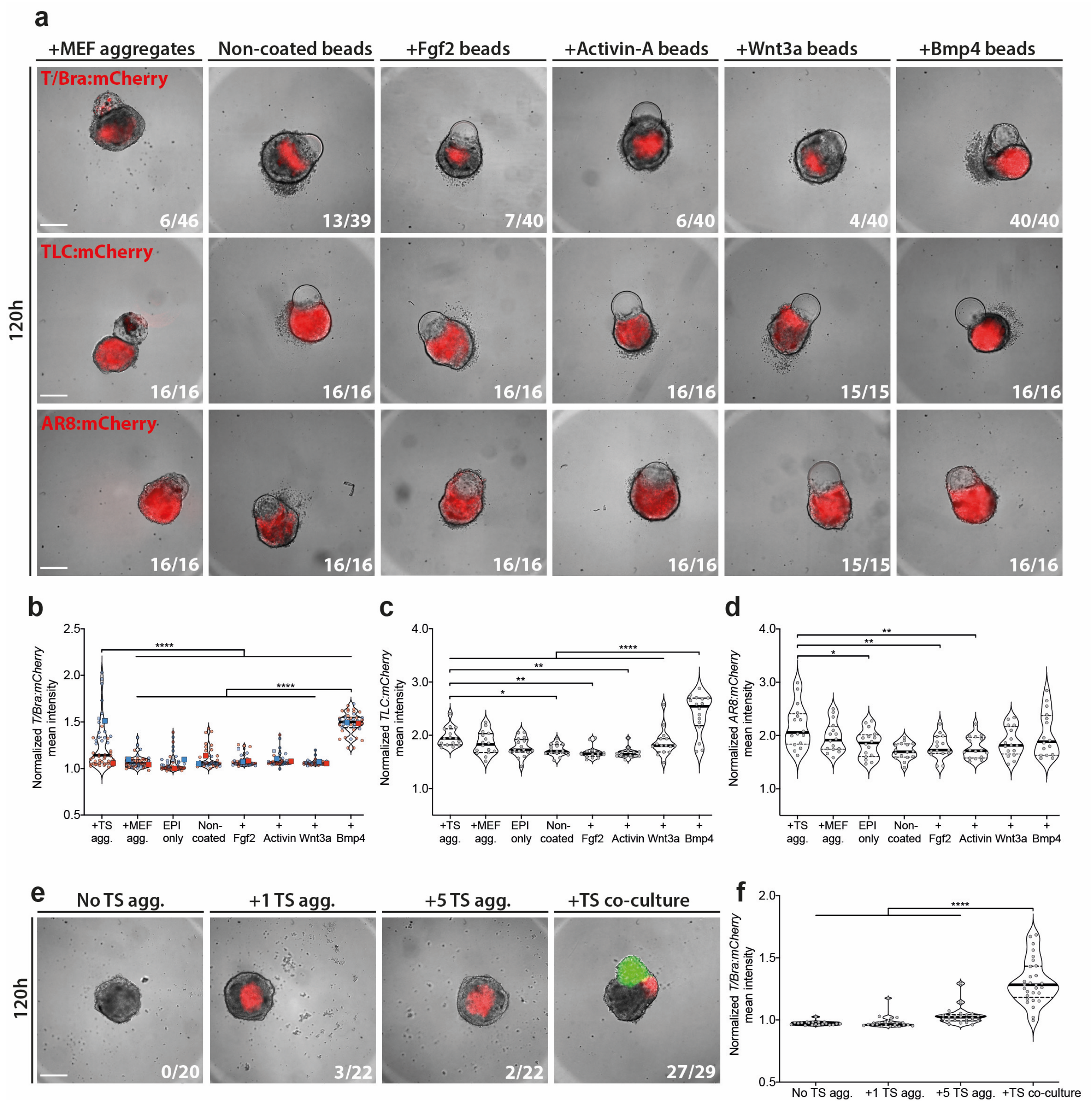

Supplementary Figure 4: Dependence of *T/Bra* expression in epithelialized EPI aggregates to TS aggregate derived signaling a) Representative images showing *T/Bra*:mCherry (top), *TLC*:mCherry (middle) and *AR8:*mCherry (bottom) reporter activities at 120h in epithelialized EPI aggregates co-cultured with mouse embryonic fibroblast (MEF) aggregates or beads coated with indicated proteins. After transfer, the medium was not changed until 120h in order to concentrate the factors. **b-d)** Quantification of background normalized mean intensities of *T/Bra*:mCherry **(b)**, *TLC*:mCherry **(c)** and *AR8:*mCherry **(d)** at 120h in epithelialized EPI aggregates co-cultured in indicated conditions. Data was collected from two independent experiments **(b)** or from single experiments **(c,d)**. For **(c),** adjusted p-values are: TS agg. vs Non-coated, *p=0.0358;* TS agg. vs Fgf2, *p=0.0047;* TS agg. vs Activin, *p=0.0030.* For **(d),** adjusted p-values are: TS agg. vs EPI only, *p=0.0402;* TS agg. vs Fgf2, *p=0.0079;* TS agg. vs Activin, *p=0.0041.* **e)** Representative images showing *T/Bra* expression in epithelialized EPI aggregates co-cultured with indicated number of TS aggregates on transwells. **f)** Quantification of background normalized mean intensity of *T/Bra*:mCherry in transwell co-culture at 120h. Data was collected from single experiment. All embryoids were formed from 100ESC/100TSC condition. For all conditions in **(a)** and **(c)**, number of *T/Bra*:mCherry -positive embryoids over total number of embryoids analyzed are indicated at bottom right. For **(b)** and **(d)**, large symbols indicate mean values of each replicate. Black lines indicate median and quartiles. For all statistical analysis, one-way

ANOVA followed by Tukey multiple comparison test was performed. Following P-value style was used: (****)<0.0001, (***) 0.0002, (**) 0.0021, (*) 0.0332, (ns) 0.1234. Scale bars: 200μm. Source data are provided as a Source Data file.

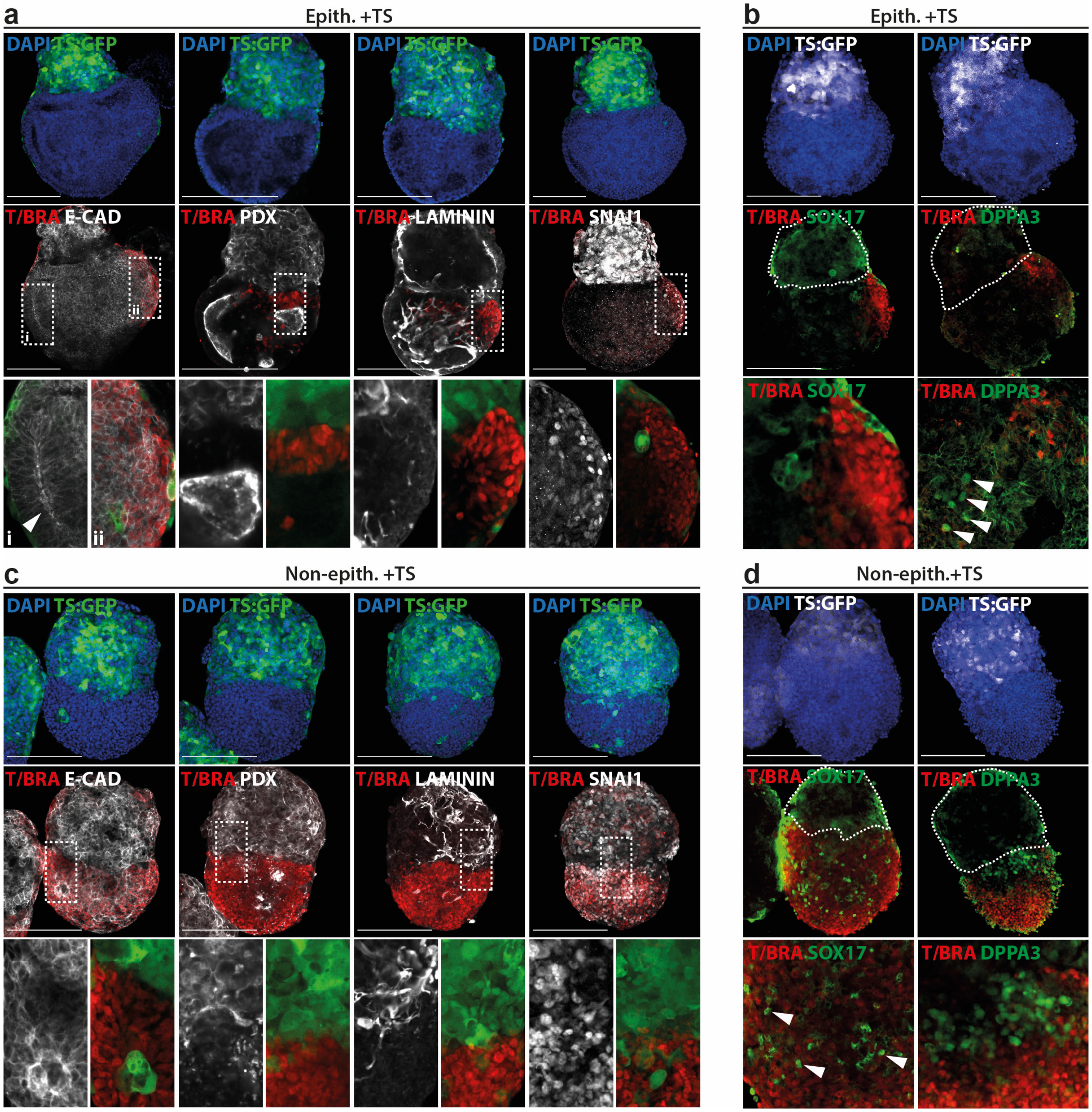

Supplementary Figure 5: Characterization of epiblast and gastrulation markers in epithelialized and non-epithelialized embryoids at 120h. a-b) Representative confocal images showing *E-cadherin, Podocalyxin, Laminin* and *Snai1* **(a)** and *Sox17*, *Dppa3* **(b)** expression in epithelialized embryoids. **c-d)** Representative confocal images showing *E-cadherin, Podocalyxin, Laminin* and *Snai1* **(c)** and *Sox17*, *Dppa3* **(d)** expression in non-epithelialized embryoids. Nuclei were stained with DAPI. GFP-labeled TS cells were depicted in green **(a,c)** or in white (**b,d**). All embryoids were formed from 100ESC/100TSC condition.

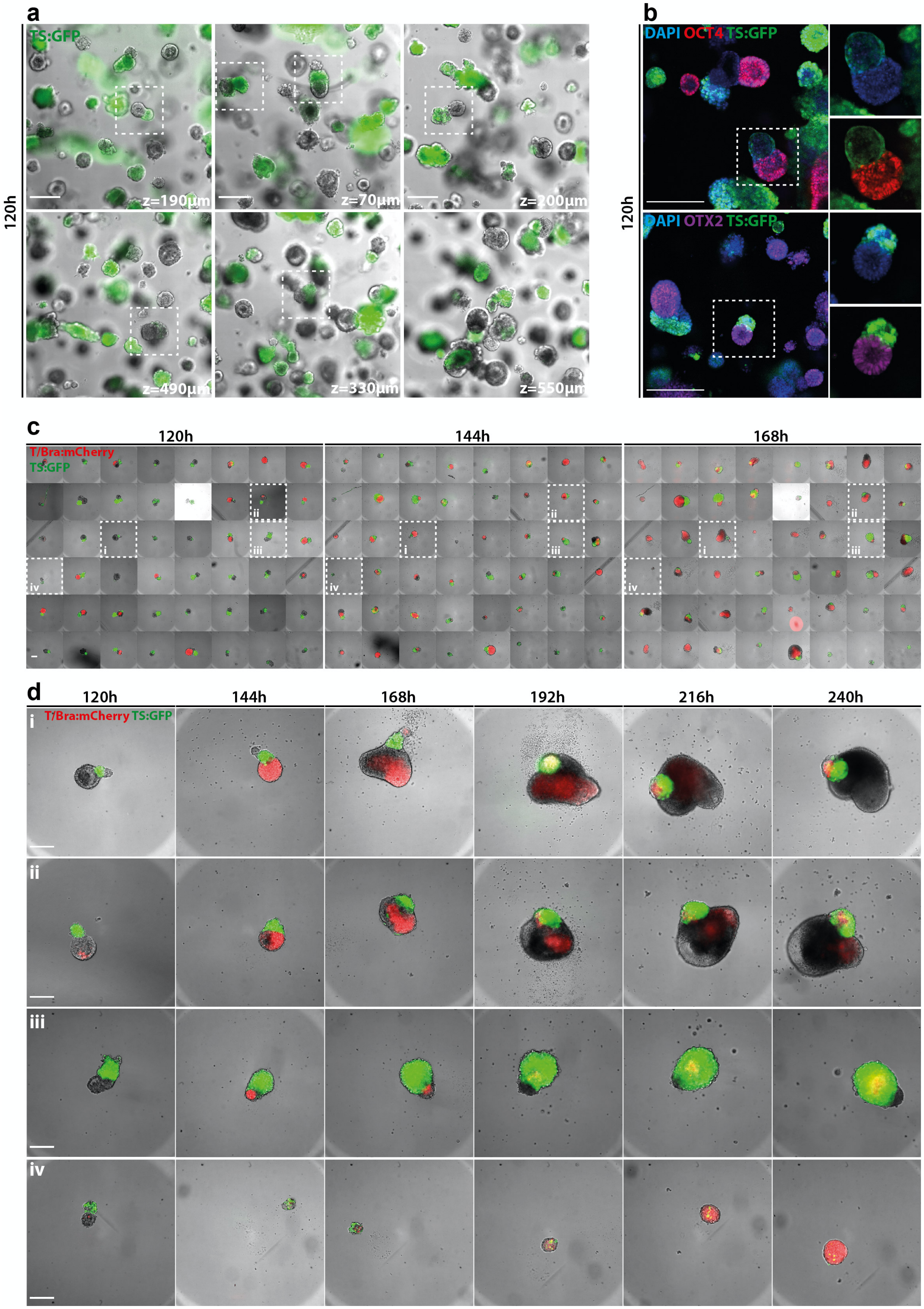

a
TS:GFP
120h
z=190µm
z=70µm
z=200µm
z=490µm
z=330µm
z=550µm
b
DAPI OCT4 TS:GFP
120h
DAPI OTX2 TS:GFP
c
120h
144h
168h
T/Bra:mCherry
TS:GFP
i
ii
iii
iv
d
120h
144h
168h
192h
216h
240h
T/Bra:mCherry TS:GFP
i
ii
iii
iv

Supplementary Figure 6: Long term culture of ETS embryos a) Representative images showing ETS embryos in culture at 120h. **b)** Representative confocal images showing *Oct4* and *Otx2* immunostainings at 120h. **c**) Montage of ETS embryos transferred to 96 well plate on 120h and cultured until 168h. **d)** Timepoint images showing extended culture until 240h and representative phenotypes.

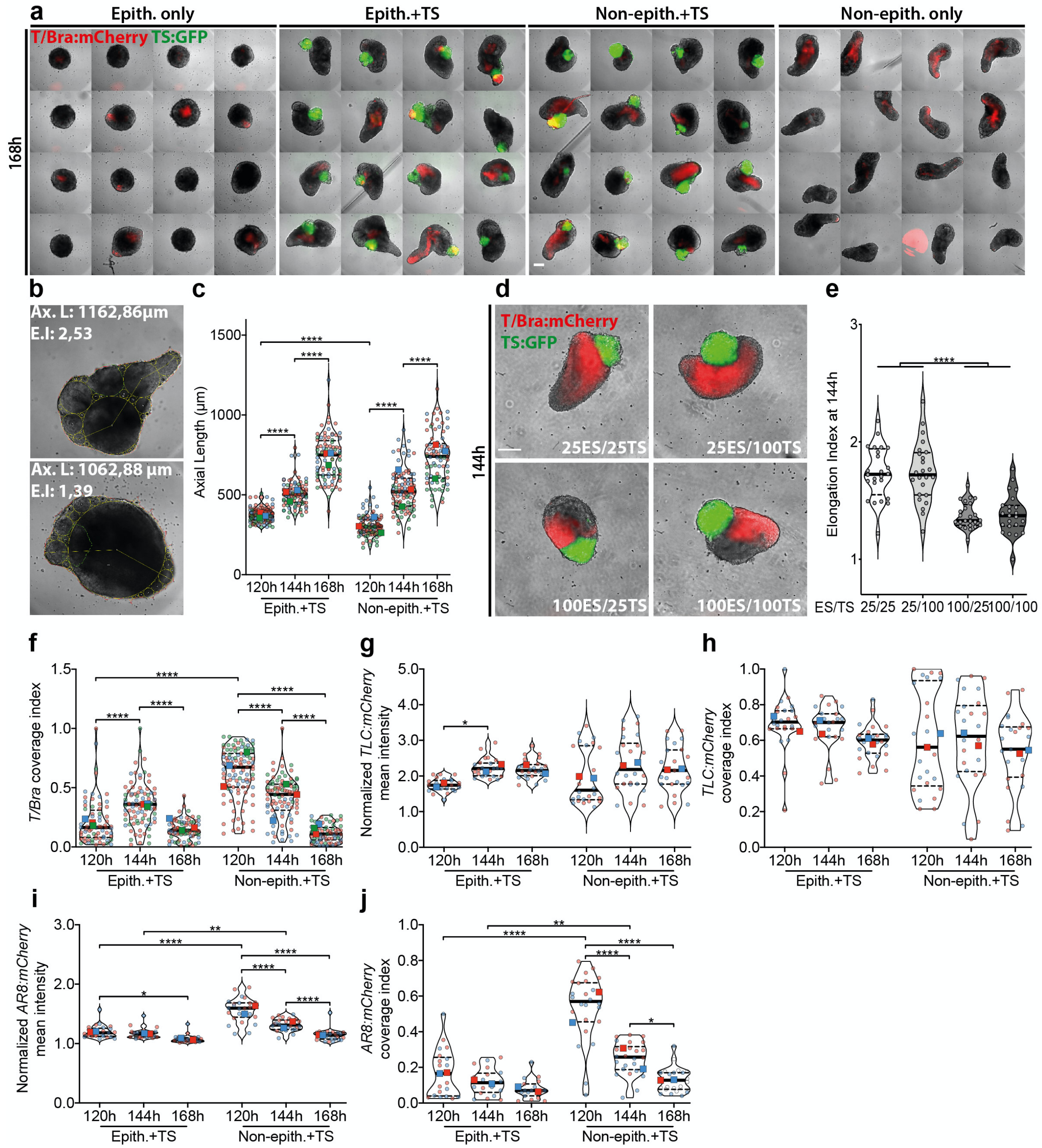

Supplementary Figure 7: Axial morphogenesis dynamics EpiTS embryoids a) Montage view of a single experiment showing reproducibility in morphology within each condition. **b)** Representative images showing axial length and elongation index calculation. **c)** Quantification of axial length of epithelialized and non-epithelialized embryoids between 120h to 168h. **d-e)** Representative images **(d)** and quantification of elongation index **(e)** of epithelialized embryoids formed from 25/25, 25/100, 100/25 and 100/100 conditions at 144h. Total number of embryoids analyzed were 24, 24, 24 and 23, respectively. For elongation index quantification, TS subtraction was performed. **f-j)** Quantification of coverage index of *T/Bra:*mCherry **(f),** *TLC:*mCherry **(h)**, *AR8:*mCherry **(j)** and background normalized *TLC:*mCherry **(g)**, *AR8:*mCherry **(i)** mean intensity in epithelialized or non-epithelialized embryoids between 120h to 168h. For **(g)**, adjusted p-values are: Epith.+TS (120h) vs Epith.+TS (144h), *p=0.0422.* For **(i)**, adjusted p-values are: Epith.+TS (120h) vs Epith.+TS (168h), *p=0.0158;* Epith.+TS (144h) vs Non-epith.+TS (144h), *p=0.0012.* For **(j)**, adjusted p-values are: Epith.+TS (144h) vs Non-epith.+TS (144h), *p=0.0065;* Non-epith.+TS (144h) vs Non-epith.+TS (168h), *p=0.0110.* All embryoids were formed from 100ESC/100TSC condition.

For all conditions in **(c,f-j)**, total number of embryoids analyzed are indicated at bottom right of **(Figure 4a-b)**. Large symbols indicate mean values of each replicate. Black lines indicate median and quartiles. For all statistical analysis, one-way ANOVA followed by Tukey multiple comparison test was performed. Following P-value style was used: (****)<0.0001, (***) 0.0002, (**) 0.0021, (*) 0.0332, (ns) 0.1234. Scale bars: 200µm. Source data are provided as a Source Data file.

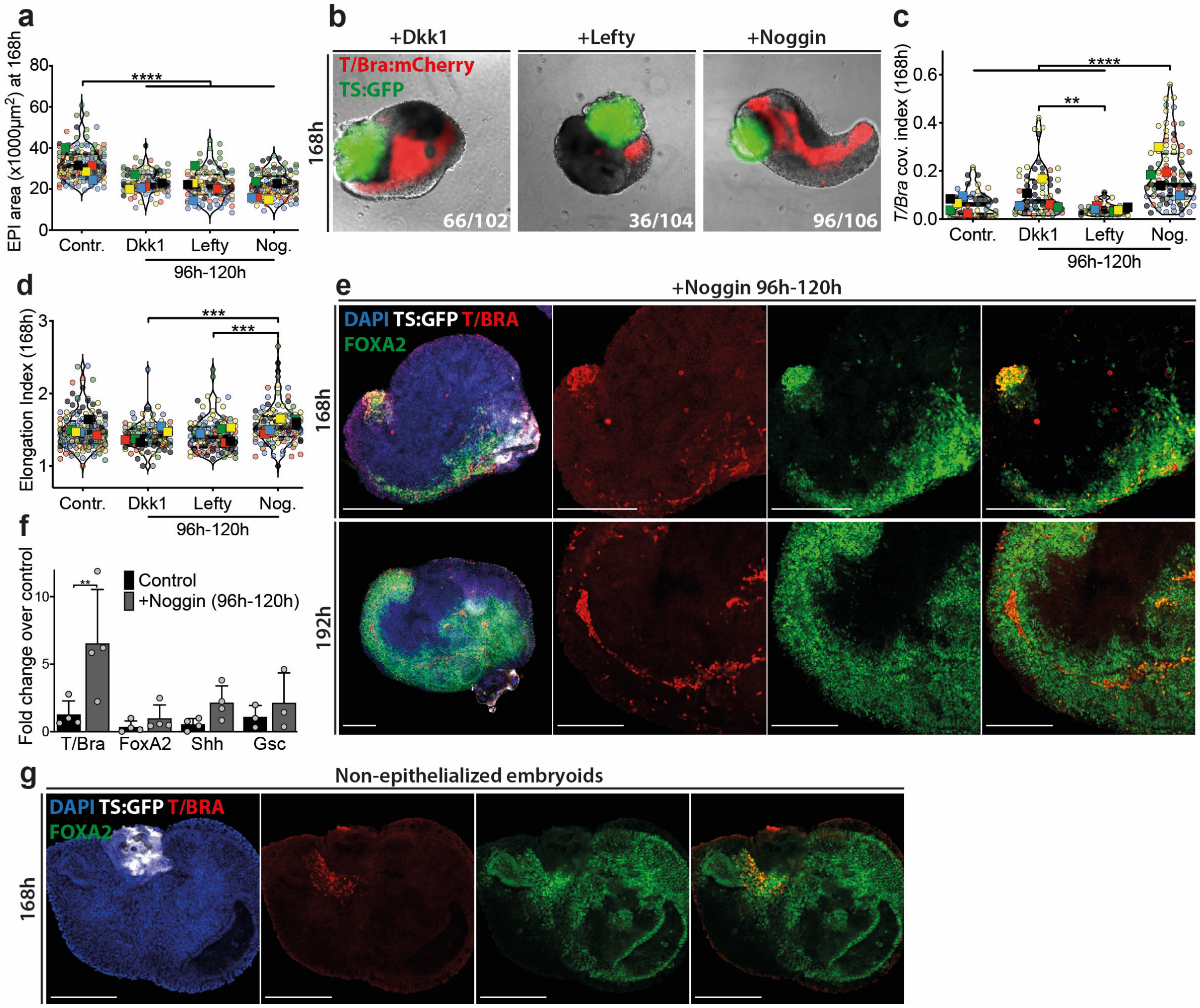

Supplementary Figure 8: Roles of WNT, TGF-β and BMP pathways in axial morphogenesis a) Quantification of EPI compartment area at 168h in epithelialized embryoids treated with indicated inhibitors between 96h-120h. **b)** Representative images at 168h showing *T/Bra:*mCherry expression in epithelialized embryoids treated with indicated inhibitors between 96h to 120h. **c-d)** Quantification of coverage index of *T/Bra:*mCherry expression **(c)** and elongation index **(d)** at 168h in epithelialized embryoids treated with indicated inhibitors. For elongation index quantification, TS subtraction was not performed. For **(c)**, adjusted p-values are: Dkk1 vs Lefty, *p=0.0060.* For **(d)**, adjusted p-values are: Lefty vs Noggin, *p=0.0003;* Dkk1 vs Noggin, *p=0.0001.* **e)** Representative confocal images of Noggin-treated epithelialized embryoid showing *T/Bra* and *Foxa2* immunostainings at 168h and 192h. **f)** RT-PCR analysis showing expression levels of *T/Bra, Foxa2, Shh* and *Gsc* in Noggin-treated epithelialized embryoids compared to untreated embryoids. Data is shown as mean. Adjusted p-values are: Control (T/Bra) vs Noggin (T/Bra), *p=0.0017.* **g)** Representative confocal images of non-epithelialized embryoids showing *T/Bra* and *Foxa2* immunostainings at 168h. Nuclei were stained with DAPI. All embryoids were formed from 100ESC/100TSC condition. For all conditions in **(a,c,d)** total number of embryoids analyzed are indicated at bottom right of **(b)**. Large symbols indicate mean values of each replicate. Black lines indicate median and quartiles. For all statistical analysis, one-way ANOVA followed by Tukey multiple comparison test was performed. Following P-value style was used: (****)<0.0001, (***) 0.0002, (**) 0.0021, (*) 0.0332, (ns) 0.1234. Scale bars: 200µm. Source data are provided as a Source Data file.

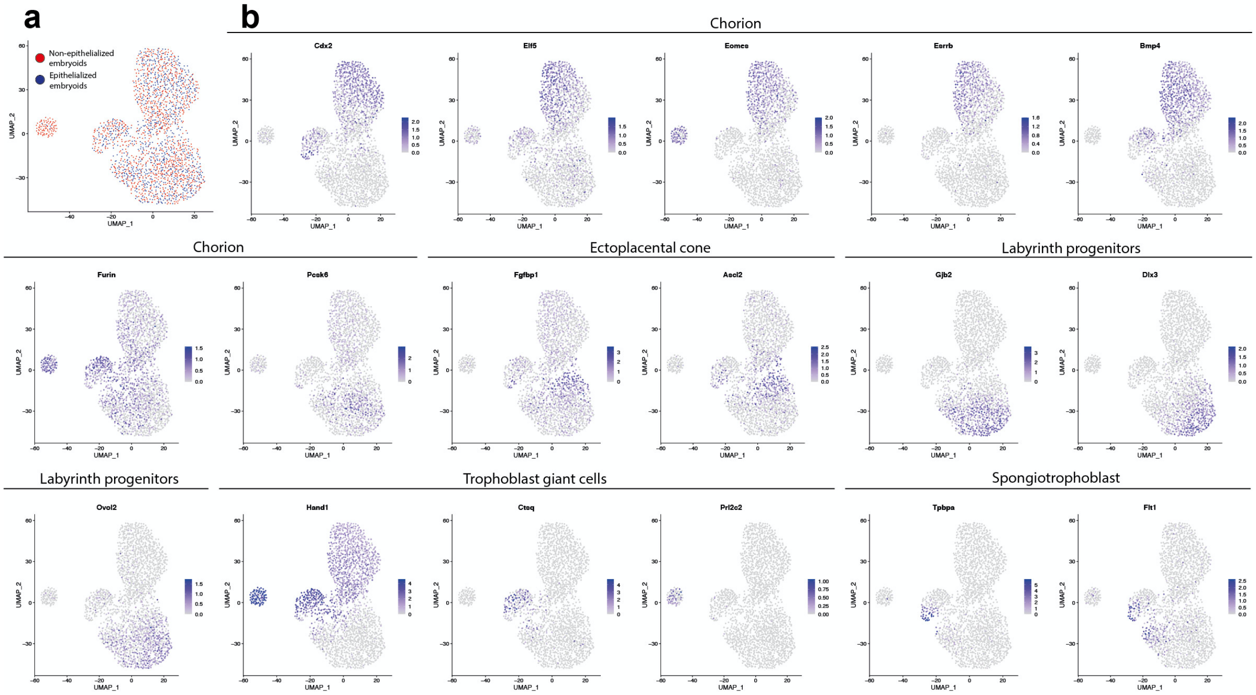

Supplementary Figure 9: Expression of key extraembryonic tissue markers in EpiTS embryoids
a) Demonstration of sample origin of cell types in extraembryonic cluster. **b)** Key markers for each extraembryonic cell type observed in EpiTS embryoids.

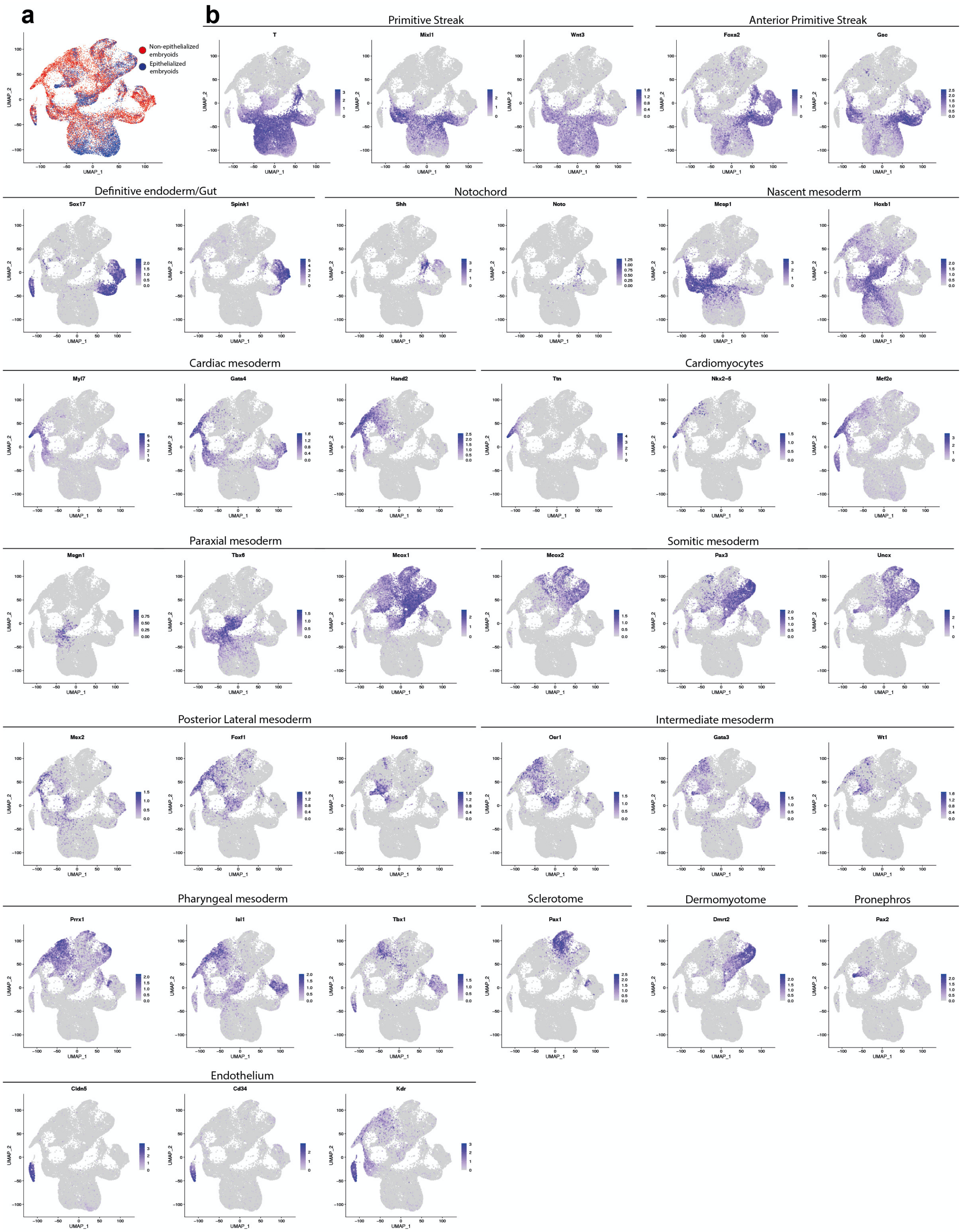

Supplementary Figure 10: Expression of key mesendoderm markers in EpiTS embryoids

a) Demonstration of sample origin of cell types in mesendoderm cluster. **b)** Key markers for each mesendoderm cell type observed in EpiTS embryoids.

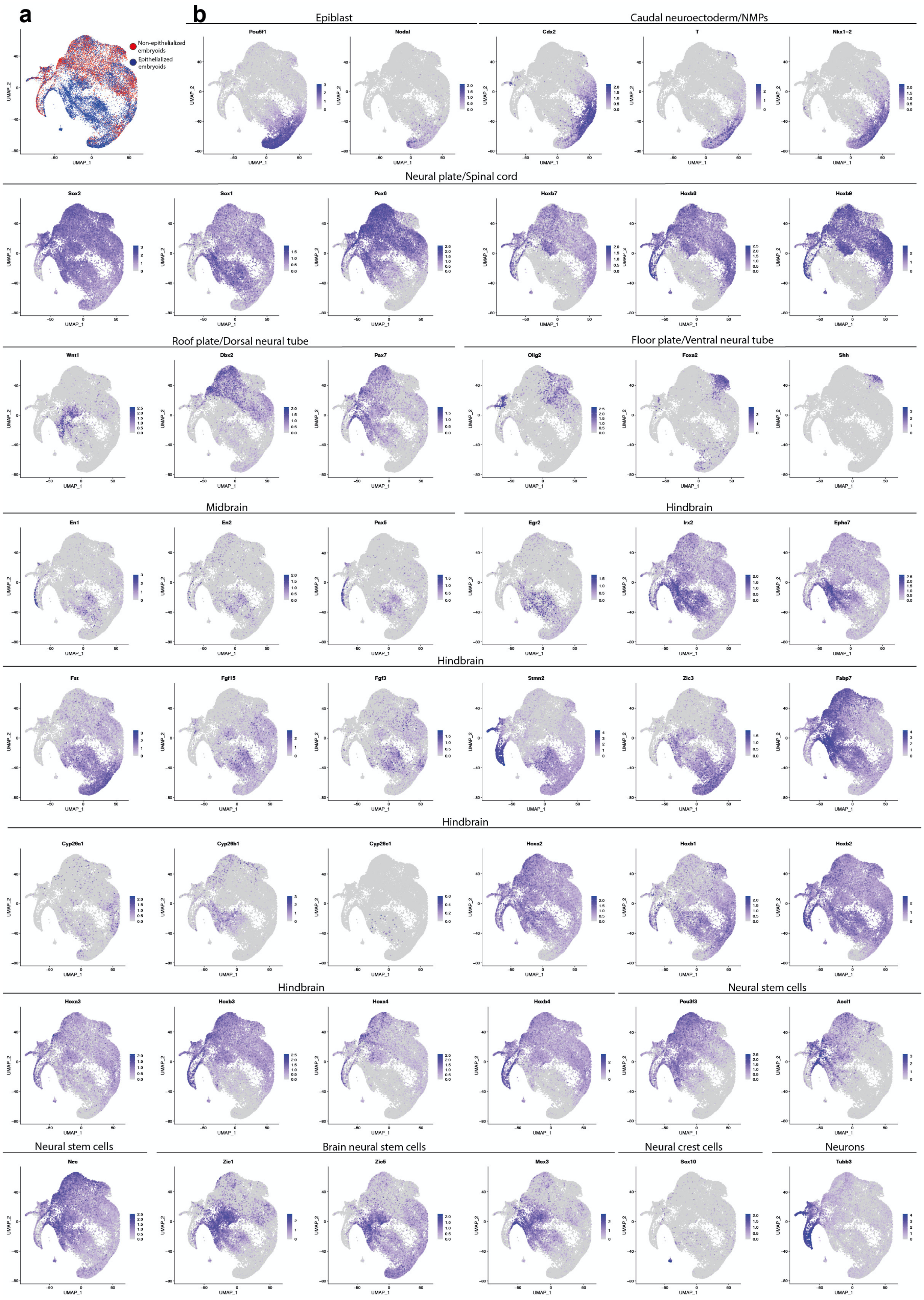
a
b
Non-epithelialized embryoids
Epithelialized embryoids
Epiblast
Pou5f1
Nodal
Caudal neuroectoderm/NMPs
Cdx2
T
Nkx1−2
Neural plate/Spinal cord
Sox2
Sox1
Pax6
Hoxb7
Hoxb8
Hoxb9
Roof plate/Dorsal neural tube
Wnt1
Dbx2
Pax7
Floor plate/Ventral neural tube
Olig2
Foxa2
Shh
Midbrain
En1
En2
Pax5
Hindbrain
Egr2
Irx2
Epha7
Hindbrain
Fst
Fgf15
Fgf3
Stmn2
Zic3
Fabp7
Hindbrain
Cyp26a1
Cyp26b1
Cyp26c1
Hoxa2
Hoxb1
Hoxb2
Hindbrain
Hoxa3
Hoxb3
Hoxa4
Hoxb4
Neural stem cells
Pou3f3
Ascl1
Neural stem cells
Nes
Brain neural stem cells
Zic1
Zic5
Mex3
Neural crest cells
Sox10
Neurons
Tubb3
UMAP_1
UMAP_2

Supplementary Figure 11: Expression of key epiblast and ectoderm markers in EpiTS embryoids
a) Demonstration of sample origin of cell types in ectoderm cluster. **b)** Key markers for each ectoderm cell type observed in EpiTS embryoids.

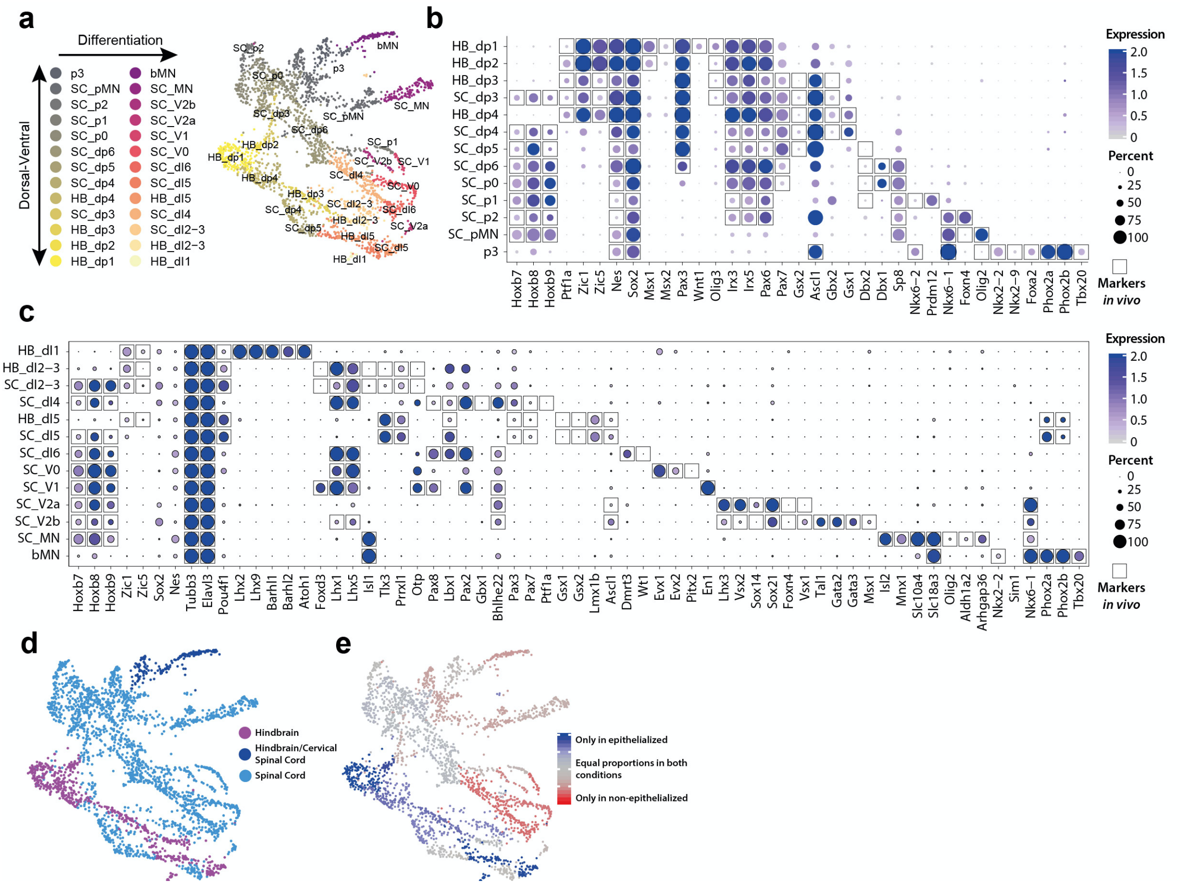

Supplementary Figure 12: Diversification of neural progenitors and neurons in EpiTS embryoids **a)** Demonstration of cell types in neurons cluster mapping to dorsal-ventral axis **b-c)** Key markers for neural progenitor **(b)** and neurons **(c)** with reference to *in vivo* atlas. **d-e)** Demonstration of tissue origin **(d)** or sample origin **(e)** of cell types in neuron cluster.

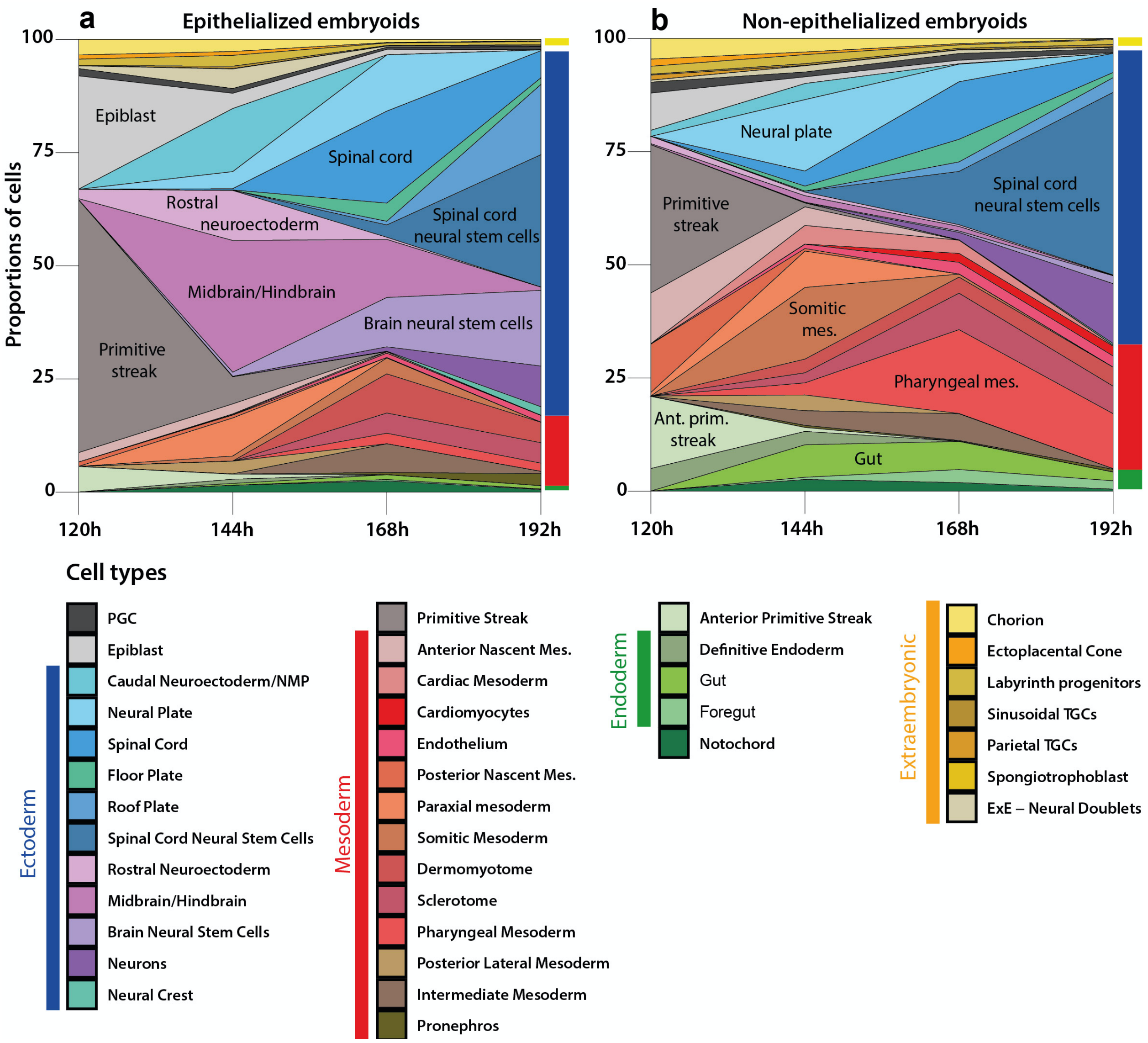

Supplementary Figure 13: Cell type proportion in epithelialized and non-epithelialized embryoids
a,b) Percentage of cells that constitute indicated cell populations between 120h to 192h in epithelialized **(a)** or non-epithelialized **(b)** embryoids.

Supplementary Table 1: List of primary antibodies used for immunostaining

Target	Species	Dilution	Catalogue Number	Supplier
anti-E-cadherin	Rabbit	1:500	#24E10	Cell Signaling Technology
anti-Podocalyxin	Rat	1:200	#MAB1556 (192703)	R&D systems
anti-Par6	Mouse	1:100	#sc-166405 (B-10)	Santa Cruz
anti- aPKC	Mouse	1:100	#sc-17781 (H-1)	Santa Cruz
anti-Sox1	Goat	1:50	#af3369	R&D Systems
anti-Sox2	Rabbit	1:400	#ab97959	Abcam
anti-Pax6	Rabbit	1:100	#901301 (Poly19013)	BioLegend
anti-Otx2	Goat	1:25	#af1979	R&D Systems
anti-Tuj1	RAbbit	1:400	#ab18207	Abcam
anti-Brachyury	Goat	1:300	#sc-17745 (C-19)	Santa Cruz
anti-Brachyury	Rabbit	1:100	#ab209665	Abcam
anti-Oct4	Mouse	1:200	#sc-5270 (C-10)	Santa Cruz
anti-Nanog	Rat	1:300	#14-5761-80	ThermoFisher
anti-Dppa3	Mouse	1:100	#AF2566-SP	R&D systems
anti-Sox17	Goat	1:200	#AF1924	Abcam
anti-Foxa2	Rabbit	1:200	#ab108422	Abcam
anti-Cdx2	Rabbit	1:200	#ab76541	Abcam
anti-Eomes	Rabbit	1:200	#ab23345	Abcam
anti-Tfap2c	Mouse	1:200	#sc-12762 (6E4/4)	Santa Cruz
anti-Six1	Rabbit	1:200	#12891S (D4A8K)	Cell Signaling Technology
anti-Eya1	Rabbit	1:100	#PA5-65034	Invitrogen
anti-Laminin	Rat	1:200	#ab44941 (LT-3)	Abcam
anti-Fibronectin	Goat	1:300	#sc-6953 (N-20)	Santa Cruz
anti-Snai1	Rabbit	1:100	#C15D3	Cell Signaling Technology
anti-mCherry	Rat	1:400	#M11217	ThermoFisher

Phalloidin AF488		1:1000	#A12379	ThermoFisher
Phalloidin AF635		1:1000	#A34054	ThermoFisher

Supplementary Table 2: List of primers used for RT-PCR

Gene	Forward Primer	Reverse Primer
Shh	GCGGCAGATATGAAGGGAAGA	CCAGGCCACTGGTTCATCAC
T/Bra	CTGGGAGCTCAGTTCTTTCG	GTCCACGAGGCTATGAGGAG
Gsc	AGACGAAGTACCCAGACGTG	CTGTCGTCTCCACTTGGCTC
FoxA2	CATTACGCCTTCAACCACCC	GGTAGTGCATGACCTGTTCG
Gapdh	CGTATTGGGCGCCTGGTCAC	ATGATGACCCTTTTGGCTCC
