# Supplementary material for: Bioengineered embryoids mimic post-implantation development in vitro
Source: Nat Commun. 2021 Aug 26;12:5140. doi: 10.1038/s41467-021-25237-8 (PMC8390504; doi:10.1038/s41467-021-25237-8)
Supplement: Supplementary file 2 — Description of Additional Supplementary Files [file 41467_2021_25237_MOESM2_ESM.docx]

**Description of Additional Supplementary Files**

**Title: Supplementary Movie 1**

**Description:** Timelapse imaging showing *T/Bra* expression dynamics until 144h in *EpiTS embryoids* formed from 25ESC/25TSC condition. Images were acquired every 2 hours. *T/Bra* expression was depicted in red, TS:GFP cells were shown in green. Scale bar: 200µm.

**Title: Supplementary Movie 2**

**Description:** Timelapse imaging showing *T/Bra* expression dynamics until 144h in *EpiTS embryoids* formed from 25ESC/100TSC condition. Images were acquired every 2 hours. *T/Bra* expression was depicted in red, TS:GFP cells were shown in green. Scale bar: 200µm.

**Title: Supplementary Movie 3**

**Description:** Timelapse imaging showing *T/Bra* expression dynamics until 144h in *EpiTS embryoids* formed from 100ESC/25TSC condition. Images were acquired every 2 hours. *T/Bra* expression was depicted in red, TS:GFP cells were shown in green. Scale bar: 200µm.

**Title: Supplementary Movie 4**

**Description:** Timelapse imaging showing *T/Bra* expression dynamics until 144h in *EpiTS embryoids* formed from 100ESC/100TSC condition. Images were acquired every 2 hours. *T/Bra* expression was depicted in red, TS:GFP cells were shown in green. Scale bar: 200µm.

**Title: Supplementary Movie 5**

**Description:** Timelapse imaging showing *T/Bra* expression dynamics until 160h in epithelialized *EpiTS embryoids* formed from 100ESC/100TSC condition. Images were acquired every 2 hours. *T/Bra* expression was depicted in red, TS:GFP cells were shown in green. Scale bar: 200µm.

**Title: Supplementary Movie 6**

**Description:** Timelapse imaging showing *T/Bra* expression dynamics until 160h in non-epithelialized *EpiTS embryoids* formed from 100ESC/100TSC condition. Images were acquired every 2 hours. *T/Bra* expression was depicted in red, TS:GFP cells were shown in green. Scale bar: 200µm.

**Title: Supplementary Movie 7**

**Description:** Timelapse imaging showing *T/Bra* expression dynamics until 168h in epithelialied *EpiTS embryoids* formed from from 100ESC/100TSC condition. Images were acquired every 2 hours. *T/Bra* expression was depicted in red, TS:GFP cells were shown in green. Scale bar: 200µm.

**Title: Supplementary Movie 8**

**Description:** Timelapse imaging showing *T/Bra* expression dynamics until 168h in epithelialied *EpiTS embryoids* formed from from 100ESC/100TSC condition and treated with 200ng/ml Dkk1 between 96h-120h. Images were acquired every 2 hours. *T/Bra* expression was depicted in red, TS:GFP cells were shown in green. Scale bar: 200µm.

**Title: Supplementary Movie 9**

**Description:** Timelapse imaging showing *T/Bra* expression dynamics until 168h in epithelialied *EpiTS embryoids* formed from from 100ESC/100TSC condition and treated with 200ng/ml LeftyA between 96h-120h. Images were acquired every 2 hours. *T/Bra* expression was depicted in red, TS:GFP cells were shown in green. Scale bar: 200µm.

**Title: Supplementary Movie 10**

**Description:** Timelapse imaging showing *T/Bra* expression dynamics until 168h in epithelialied *EpiTS embryoids* formed from from 100ESC/100TSC condition and treated with 200ng/ml Noggin between 96h-120h. Images were acquired every 2 hours. *T/Bra* expression was depicted in red, TS:GFP cells were shown in green. Scale bar: 200µm.
