# Supplementary material for: Bioengineered embryoids mimic post-implantation development in vitro
Source: Nat Commun. 2021 Aug 26;12:5140. doi: 10.1038/s41467-021-25237-8 (PMC8390504; doi:10.1038/s41467-021-25237-8)
Supplement: Supplementary file 13 — Reporting Summary [file 41467_2021_25237_MOESM13_ESM.pdf]

natureresearch

Corresponding author(s): Matthias Lutolf

Last updated by author(s): May 18, 2021

Reporting Summary

Nature Research wishes to improve the reproducibility of the work that we publish. This form provides structure for consistency and transparency in reporting. For further information on Nature Research policies, see Authors & Referees and the Editorial Policy Checklist .

Statistics

For all statistical analyses, confirm that the following items are present in the figure legend, table legend, main text, or Methods section.

n/a	Confirmed	
☐	☒	The exact sample size (*n*) for each experimental group/condition, given as a discrete number and unit of measurement
☐	☒	A statement on whether measurements were taken from distinct samples or whether the same sample was measured repeatedly
☐	☒	The statistical test(s) used AND whether they are one- or two-sided<br>*Only common tests should be described solely by name; describe more complex techniques in the Methods section.*
☐	☒	A description of all covariates tested
☐	☒	A description of any assumptions or corrections, such as tests of normality and adjustment for multiple comparisons
☐	☒	A full description of the statistical parameters including central tendency (e.g. means) or other basic estimates (e.g. regression coefficient) AND variation (e.g. standard deviation) or associated estimates of uncertainty (e.g. confidence intervals)
☒	☐	For null hypothesis testing, the test statistic (e.g. *F*, *t*, *r*) with confidence intervals, effect sizes, degrees of freedom and *P* value noted<br>*Give P values as exact values whenever suitable.*
☒	☐	For Bayesian analysis, information on the choice of priors and Markov chain Monte Carlo settings
☒	☐	For hierarchical and complex designs, identification of the appropriate level for tests and full reporting of outcomes
☒	☐	Estimates of effect sizes (e.g. Cohen's *d*, Pearson's *r*), indicating how they were calculated

Our web collection on statistics for biologists contains articles on many of the points above.

Software and code

Policy information about availability of computer code

Data collection	NIS Elements (Nikon Corporation), ZEN 2010 (Zeiss AG), QuantStudio 6(Applied Biosystems), NanoDrop 2000/2000c (Thermo Scientific)
Data analysis	Fiji (v2.0.0-rc-69/1.52p) and custom plugins for image processing from Bioimaging Core Facility(BIOP, EPFL), Microsoft Excel 2011 (v14.5.5.) (Microsoft Corporation), GraphPad Prism (v9.1.2), Cutadapt (v2.1) , Seurat (v3.1)

For manuscripts utilizing custom algorithms or software that are central to the research but not yet described in published literature, software must be made available to editors/reviewers. We strongly encourage code deposition in a community repository (e.g. GitHub). See the Nature Research guidelines for submitting code & software for further information.

Data

Policy information about availability of data

All manuscripts must include a data availability statement. This statement should provide the following information, where applicable:
- Accession codes, unique identifiers, or web links for publicly available datasets
- A list of figures that have associated raw data
- A description of any restrictions on data availability

All data regarding image processing/quantification and RNA-se uencing analysis are available upon request.

Field-specific reporting

Please select the one below that is the best fit for your research. If you are not sure, read the appropriate sections before making your selection.

☒ Life sciences ☐ Behavioural & social sciences ☐ Ecological, evolutionary & environmental sciences

Life sciences study design

All studies must disclose on these points even when the disclosure is negative.

Sample size
For EPI and TSC aggregates generated on microwells at 72 h, the sample size was >100. After transferring aggregates to 96 well plates, in general, the experiment design was to set 4 conditions with 24 aggregates per condition.

Data exclusions
EpiTS embryoids that accidentally contain more than one EPI or TSC aggregate were excluded from the analysis. Also, EpiTS embryoids lost during medium exchange were excluded.

Replication
All experiments were successfully replicated at least 3 times with similar results.

Randomization
EPI and TSC aggregates with rounded morphology and no clear sign of apoptosis were picked and transfered together to form EpiTS embryoids.

Blinding
Blinding was not performed.

Reporting for specific materials, systems and methods

We require information from authors about some types of materials, experimental systems and methods used in many studies. Here, indicate whether each material, system or method listed is relevant to your study. If you are not sure if a list item applies to your research, read the appropriate section before selecting a response.

Materials & experimental systems

n/a	Involved in the study
☐	☒ Antibodies
☐	☒ Eukaryotic cell lines
☒	☐ Palaeontology
☒	☐ Animals and other organisms
☒	☐ Human research participants
☒	☐ Clinical data

Methods

n/a	Involved in the study
☒	☐ ChIP-seq
☒	☐ Flow cytometry
☒	☐ MRI-based neuroimaging

Antibodies

Antibodies used
Primary antibodies used in this study:
anti-E-cadherin Rabbit 1:500 #24E10 Cell Signaling Technology
anti-Sox2 Rabbit 1:400 #ab97959 Abcam
anti-Sox1 Goat 1:50 #af3369 R&D Systems
anti-Otx2 Goat 1:25 #af1979 R&D Systems
anti-Brachyury Goat 1:300 #sc-17745 (C-19) Santa Cruz
anti-Brachyury Rabbit 1:100 #ab209665 Abcam
anti-Oct4 Mouse 1:200 #sc-5270 (C-10) Santa Cruz
anti-Nanog Rat 1:300 #14-5761-80 ThermoFisher
anti-Cdx2 Rabbit 1:200 #ab76541 Abcam
anti-Eomes Rabbit 1:200 #ab23345 Abcam
anti-aPKC Mouse 1:100 #sc-17781 (H-1) Santa Cruz
anti-Pax6 Rabbit 1:100 #901301 (Poly19013) BioLegend
anti-Six1 Rabbit 1:200 #12891S (D4A8K) Cell Signaling Technology
anti-Podocalyxin Rat 1:200 #MAB1556 (192703) R&D systems
anti-Par6 Mouse 1:100 #sc-166405 (B-10) Santa Cruz
anti-Tuj1 RAbbit 1:400 #ab18207 Abcam
anti-Dppa3 Mouse 1:100 #AF2566-SP R&D systems
anti-Sox17 Goat 1:200 #AF1924 Abcam
anti-Foxa2 Rabbit 1:200 #ab108422 Abcam
anti-Tfap2c Mouse 1:200 #sc-12762 (6E4/4) Santa Cruz
anti-Eya1 Rabbit 1:100 #PA5-65034 Invitrogen
anti-Laminin Rat 1:200 #ab44941 (LT-3) Abcam
anti-Fibronectin Goat 1:300 #sc-6953 (N-20) Santa Cruz
anti-Snai1 Rabbit 1:100 #C15D3 Cell Signaling Technology
anti-mCherry Rat 1:400 #M11217 ThermoFisher

Secondary antibodies used in this study were provided by Histology Core facility at EPFL

Validation
Antibodies for Brachyury, Sox17 and Cdx2 were validated in Beccari et. al and Turner et. al.
Antibodies for Sox2 and Oct4 were validated in Manfrin et. al.
E-cadherin antibody was validated by the manifacturer for immunostaining of MCF7 cells.
Sox1 antibody was validated by the manifacturer for immunostaining of Mouse Cortical Stem Cells.
Otx2 antibody was validated by the manifacturer for immunostaining of NTera-2 Human Cell Line.
Nanog antibody was validated by the manifacturer for immunostaining of F9 Cell Line.
Eomes antibody was validated by the manifacturer for immunostaining of adult mouse SVZ (subventricular zone).
aPKC antibody was validated by the manifacturer for immunostaining of SW480 cells.
Pax6 antibody was validated by Milad Riazifar (U.C. Irvine) for immunostaining of human iPSC derived neural rosettes.Ee have
Six1 antibody was validated by the manifacturer for immunostaining of A204 cells.
Podocalyxin antibody was validated by the manifacturer for immunostaining of bEnd.3 Mouse Cell Line.
Par6 antibody was validated by the manifacturer for immunostaining of human duodenum tissue.
Tuj1 antibody was validated by the manifacturer for immunostaining of SK-N-SH (Human neuroblastoma cell line) cells.
Dppa3 antibody was validated by the manifacturer for immunostaining of mouse ovary tissue.
Foxa2 antibody was validated by the manifacturer for immunostaining of HT-29 cells.
Tfap2c antibody was validated by the manifacturer for immunostaining of HeLa cells.
Eya1 antibody was validated by the manifacturer for immunostaining of RH-30 cells.
Laminin antibody was validated by the manifacturer for immunostaining of LS174T cells.
Fibronectin antibody was validated by Früh et. al., 2015 for immunostaining of Normal Human Dermal Fibroblasts.
Snai1 antibody was validated by Kunnen et. al., 2017 for immunostaining of Proximal Tubular Epithelial Cells (PTECs).
mCherry antibody was validated by the manifacturer for immunostaining of mCherry transduced U2OS cells.

Eukaryotic cell lines

Policy information about cell lines

Cell line source(s)	SBR ES cell line was generated and provided by David Suter Lab (Deluz et. al., 2016)<br>TLC:mCherry line was generated by Ferrer Vaquer et. al., 2010 and provided by Alfonso Martinez-Arias.<br>AR8:mCherry line was generated by Serup et. al., 2012 and provided by Alfonso Martinez-Arias.<br>TS:GFP cells was generated by Tanaka et. al., 1998 and provided by Christian Schröter.
Authentication	SBR, TLC:mCherry and AR8:mCherry cell lines were authenticated by PCR genotyping following gene targeting.<br>TS:GFP cell line was authenticated in Tanaka et. al., Science, 1998. The authentication was done by chimera formation and GFP detection in only extraembryonic tissues.
Mycoplasma contamination	All cell lines were tested regularly and confirmed free of mycoplasma with in house mycoplasma test and MYCOPLASMACHECK service from GATC.
Commonly misidentified lines<br>(See ICLAC register)	No cell lines used in this study are in the data-base of commonly misidentified cell lines.
